# AKG/OXGR1 promotes skeletal muscle blood flow and metabolism by relaxing vascular smooth muscle

**DOI:** 10.1093/lifemeta/loac026

**Published:** 2022-09-29

**Authors:** Jinping Yang, Guli Xu, Yiming Xu, Pei Luo, Yexian Yuan, Lin Yao, Jingjing Zhou, Yunlong Zhu, Ishwari Gyawali, Chang Xu, Jinlong Feng, Zewei Ma, Yuxian Zeng, Songbo Wang, Ping Gao, Canjun Zhu, Qingyan Jiang, Gang Shu

**Affiliations:** Guangdong Laboratory for Lingnan Modern Agriculture and Guangdong Province Key Laboratory of Animal Nutritional Regulation, South China Animal Nutrition and Feed Science Observation and Experimental Station, College of Animal Science, South China Agricultural University, 483 Wushan Road, Tianhe District, Guangzhou, Guangdong 510642, China; Guangdong Laboratory for Lingnan Modern Agriculture and Guangdong Province Key Laboratory of Animal Nutritional Regulation, South China Animal Nutrition and Feed Science Observation and Experimental Station, College of Animal Science, South China Agricultural University, 483 Wushan Road, Tianhe District, Guangzhou, Guangdong 510642, China; School of Basic Medical Sciences, The Sixth Affiliated Hospital of Guangzhou Medical University, Qingyuan People’s Hospital, Guangzhou Medical University, Guangzhou, Guangdong 511518, China; Guangdong Laboratory for Lingnan Modern Agriculture and Guangdong Province Key Laboratory of Animal Nutritional Regulation, South China Animal Nutrition and Feed Science Observation and Experimental Station, College of Animal Science, South China Agricultural University, 483 Wushan Road, Tianhe District, Guangzhou, Guangdong 510642, China; Guangdong Laboratory for Lingnan Modern Agriculture and Guangdong Province Key Laboratory of Animal Nutritional Regulation, South China Animal Nutrition and Feed Science Observation and Experimental Station, College of Animal Science, South China Agricultural University, 483 Wushan Road, Tianhe District, Guangzhou, Guangdong 510642, China; South China Research Center for Acupuncture and Moxibustion, Medical College of Acu-Moxi and Rehabilitation, Guangzhou University of Chinese Medicine, Guangzhou, Guangdong 510006, China; School of Pharmaceutical Sciences, Guangzhou University of Chinese Medicine, Guangzhou, Guangdong 510006, China; Guangdong Laboratory for Lingnan Modern Agriculture and Guangdong Province Key Laboratory of Animal Nutritional Regulation, South China Animal Nutrition and Feed Science Observation and Experimental Station, College of Animal Science, South China Agricultural University, 483 Wushan Road, Tianhe District, Guangzhou, Guangdong 510642, China; Guangdong Laboratory for Lingnan Modern Agriculture and Guangdong Province Key Laboratory of Animal Nutritional Regulation, South China Animal Nutrition and Feed Science Observation and Experimental Station, College of Animal Science, South China Agricultural University, 483 Wushan Road, Tianhe District, Guangzhou, Guangdong 510642, China; Guangdong Laboratory for Lingnan Modern Agriculture and Guangdong Province Key Laboratory of Animal Nutritional Regulation, South China Animal Nutrition and Feed Science Observation and Experimental Station, College of Animal Science, South China Agricultural University, 483 Wushan Road, Tianhe District, Guangzhou, Guangdong 510642, China; Guangdong Laboratory for Lingnan Modern Agriculture and Guangdong Province Key Laboratory of Animal Nutritional Regulation, South China Animal Nutrition and Feed Science Observation and Experimental Station, College of Animal Science, South China Agricultural University, 483 Wushan Road, Tianhe District, Guangzhou, Guangdong 510642, China; Guangdong Laboratory for Lingnan Modern Agriculture and Guangdong Province Key Laboratory of Animal Nutritional Regulation, South China Animal Nutrition and Feed Science Observation and Experimental Station, College of Animal Science, South China Agricultural University, 483 Wushan Road, Tianhe District, Guangzhou, Guangdong 510642, China; Guangdong Laboratory for Lingnan Modern Agriculture and Guangdong Province Key Laboratory of Animal Nutritional Regulation, South China Animal Nutrition and Feed Science Observation and Experimental Station, College of Animal Science, South China Agricultural University, 483 Wushan Road, Tianhe District, Guangzhou, Guangdong 510642, China; Guangdong Laboratory for Lingnan Modern Agriculture and Guangdong Province Key Laboratory of Animal Nutritional Regulation, South China Animal Nutrition and Feed Science Observation and Experimental Station, College of Animal Science, South China Agricultural University, 483 Wushan Road, Tianhe District, Guangzhou, Guangdong 510642, China; Guangdong Laboratory for Lingnan Modern Agriculture and Guangdong Province Key Laboratory of Animal Nutritional Regulation, South China Animal Nutrition and Feed Science Observation and Experimental Station, College of Animal Science, South China Agricultural University, 483 Wushan Road, Tianhe District, Guangzhou, Guangdong 510642, China; Guangdong Laboratory for Lingnan Modern Agriculture and Guangdong Province Key Laboratory of Animal Nutritional Regulation, South China Animal Nutrition and Feed Science Observation and Experimental Station, College of Animal Science, South China Agricultural University, 483 Wushan Road, Tianhe District, Guangzhou, Guangdong 510642, China; Guangdong Laboratory for Lingnan Modern Agriculture and Guangdong Province Key Laboratory of Animal Nutritional Regulation, South China Animal Nutrition and Feed Science Observation and Experimental Station, College of Animal Science, South China Agricultural University, 483 Wushan Road, Tianhe District, Guangzhou, Guangdong 510642, China; Guangdong Laboratory for Lingnan Modern Agriculture and Guangdong Province Key Laboratory of Animal Nutritional Regulation, South China Animal Nutrition and Feed Science Observation and Experimental Station, College of Animal Science, South China Agricultural University, 483 Wushan Road, Tianhe District, Guangzhou, Guangdong 510642, China; Guangdong Laboratory for Lingnan Modern Agriculture and Guangdong Province Key Laboratory of Animal Nutritional Regulation, South China Animal Nutrition and Feed Science Observation and Experimental Station, College of Animal Science, South China Agricultural University, 483 Wushan Road, Tianhe District, Guangzhou, Guangdong 510642, China

**Keywords:** OXGR1, AKG, smooth muscle cell, skeletal muscle, blood flow, metabolism

## Abstract

In response to contraction during exercise, skeletal muscle growth and metabolism are dynamically regulated by nerve action, blood flow, and metabolic feedback. *α*-Ketoglutarate (AKG), a bioactive intermediate in the tricarboxylic acid cycle released during exercise, has been shown to promote skeletal muscle hypertrophy. However, the underlying mechanism of AKG in regulating skeletal muscle development and metabolism is still less known. 2-Oxoglutarate receptor 1 (OXGR1), the endogenous AKG receptor, is found to be distributed in the vascular smooth muscle (VSM) of skeletal muscles. OXGR1 knockout results in skeletal muscle atrophy, accompanied by decreased expression of myosin heavy chain I (MyHC I), capillary density, and endurance exercise capacity. Furthermore, the study found that dietary AKG supplementation increased mice endurance exercise distance, MyHC I/MyHC IIb ratio, arteriole, and capillary densities in skeletal muscle. Meanwhile, acute AKG administration gradually increased the blood flow in the lower limbs. Further, by using OXGR1 global knockout and OXGR1 VSM-specific (MYH11-Cre × OXGR1-FloxP) knockdown models, we found that OXGR1 in VSM is essential for AKG-induced improvement of skeletal muscle performances. According to the *in vitro* study, AKG expanded the cell area in VSM with a decreased intracellular pH by OXGR1. Our results demonstrated a novel role of AKG/OXGR1 in VSM of skeletal muscle to regulate blood flow and then enhance slow muscle fiber conversion and capillarization. These findings provide a theoretical basis for the AKG/OXGR1 signaling pathway to maintain human muscle function and improve meat production and livestock and poultry meat quality.

## Introduction

Skeletal muscle, comprising around 40% of body mass, is a heterogeneous tissue consisting of various fibers characterized by different contractile and metabolic properties [1]. Muscle mass and metabolism are essential for human health. Simultaneously, they also affect the quality and production of meat of livestock and poultry. The growth and metabolism of skeletal muscles are influenced by numerous factors, including nerve regulation, hormone secretion, and blood flow. The increase in blood flow promotes angiogenesis [2, 3], a process that affects muscle fiber metabolism and type conversion.

Besides neuroendocrine factors, other local metabolites, such as nitric oxide, purine, and amino acids, also affect skeletal muscle blood supply and angiogenesis [4, 5]. α-Ketoglutarate (AKG) is an essential metabolite of the tricarboxylic acid (TCA) cycle and glutamate. The physiological blood level of AKG increases significantly after resistance exercise, but the increase extent gradually decreases during aging [6]. There are a number of studies that have demonstrated the significance of AKG as a metabolic signaling molecule with important biological activity. For muscle development, it has been established that AKG promotes the synthesis of muscle protein and the development of muscle metabolism in mice [6], chicken [7], and pigs [8]. However, the principal mechanism of AKG in regulating skeletal muscle development and metabolism has not been fully understood yet.

As the cell membrane is impermeable to AKG, the effects of extracellular AKG are mostly mediated by its receptor 2-oxoglutarate receptor (OXGR1), while the OXGR1 mRNA expression is lower in skeletal muscle than in other tissues, such as the testis and kidneys [9]. Interestingly, in our previous study, we discovered that OXGR1 knockout caused dramatic skeletal muscle atrophy in mice, which shows the key role of OXGR1 in skeletal muscle development. Bankova *et al*. found that OXGR1 is expressed in the bronchial smooth muscle and regulates the trachea diastolic [10]. This evidence leads to a hypothesis that OXGR1 may also locate smooth muscle-like cells in the skeletal muscle, thereby regulating skeletal muscle growth and metabolism.

First, this study demonstrated that OXGR1 is expressed in vascular smooth muscle (VSM) in skeletal muscle by immunofluorescence co-localization. Then, both the AKG ligand pharmacological and OXGR1 knockout genetic models revealed that OXGR1 plays a significant role in skeletal muscle blood supply, capillary density, muscle metabolism, and fiber type transition. As per the *in vitro* study, the AKG/OXGR1 induced smooth muscle cell relaxation by calcium signaling and intracellular acid–base balance. This study discloses a novel role of the AKG/OXGR1 in VSM relaxation and skeletal muscle metabolism, which offers new ideas for AKG as a nutrient or medicine to maintain human muscle function, increase meat production, and enhance meat quality in livestock and poultry industry.

## Results

### Skeletal muscle OXGR1 is expressed in VSM

**Figure 1:**
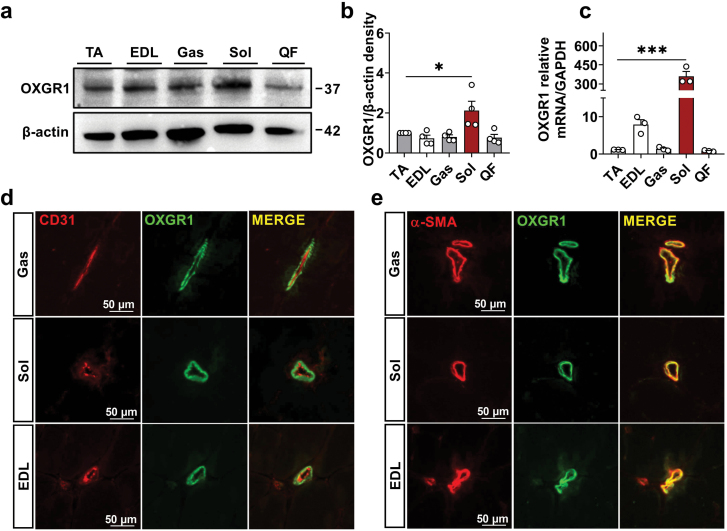
The distribution of OXGR1 in skeletal muscle. (a and b) The protein expression of OXGR1 in the skeletal muscle of C57BL/6J mice (*n* = 4). (c) The mRNA expression of OXGR1 in the skeletal muscle of C57BL/6J mice (*n* = 3). (d and e) Co-immunolocalization of OXGR1 (green) and vascular endothelial cells (CD31 in red) or OXGR1 and vascular smooth muscle cells (α-SMA in red) in skeletal muscle of mice (*n* = 3). Data information: results are presented as mean ± SEM. In (a) and (b), different groups were analyzed for statistical significance by one-way ANOVA; *P ≤ 0.05, ***P ≤ 0.001.

To address if OXGR1 is expressed in the skeletal muscle, we first compared the protein expression levels of OXGR1 in different muscles of C57BL/6J mice, the Tibialis Anterior, Gastrocnemius, Soleus, Extensor Digitorum Longus, and Quadriceps Femoris. The findings revealed that OXGR1 was highly expressed in the Soleus, rich in blood vessels (Fig. 1a−c). Afterward, we investigated the cellular distribution of OXGR1 in skeletal muscle. The immunofluorescence study established that OXGR1 was co-localized with *α*-SMA, a VSM marker, but not the CD31, the vascular endothelial marker (Fig. 1d and e). This evidence indicated that skeletal OXGR1 is present in VSM and highly expressed in oxidative red muscle.

### OXGR1 is required for maintaining the proportion of oxidative red muscle and capillary density in skeletal muscle

**Figure 2:**
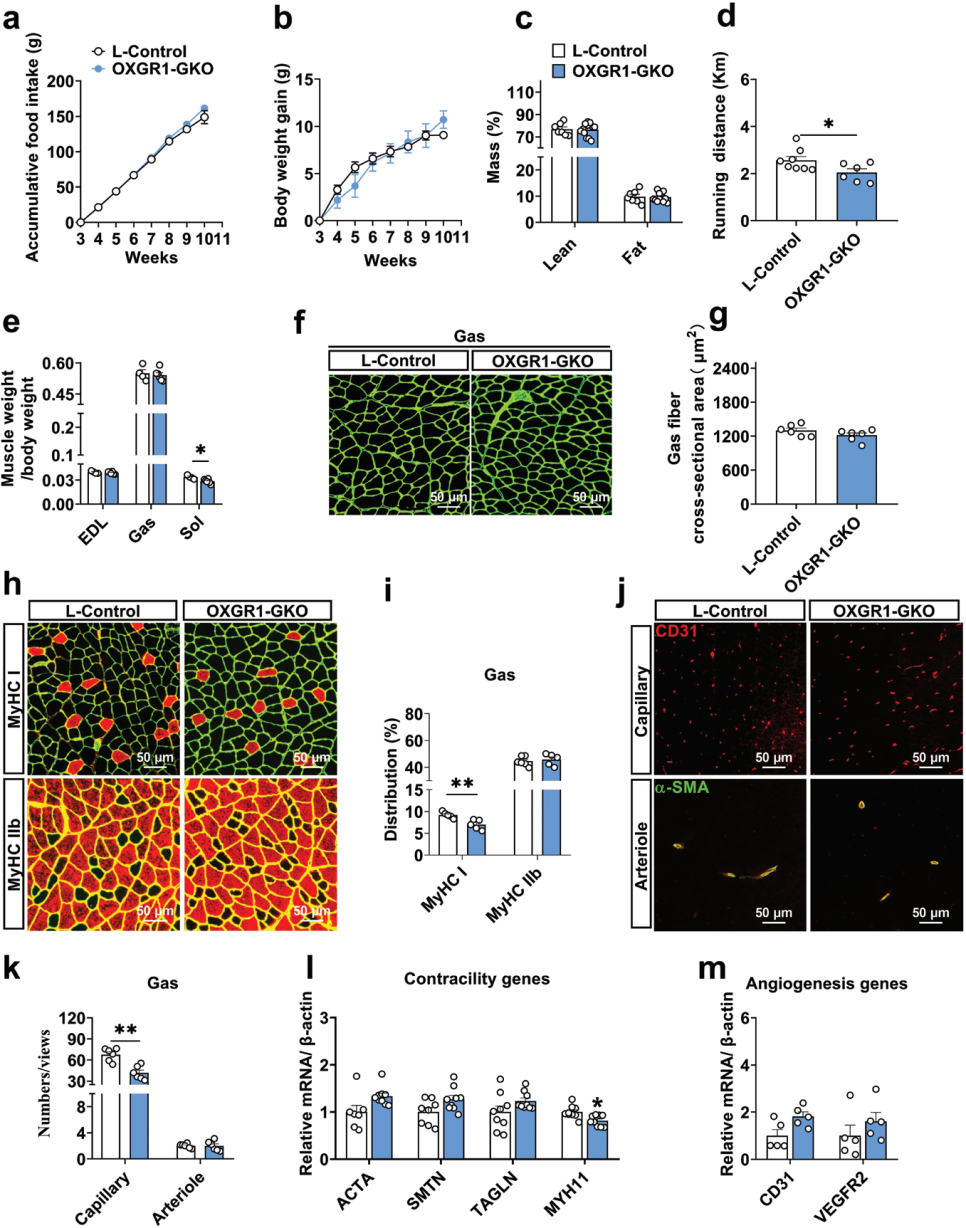
OXGR1 knockout impairs skeletal muscle function, fiber type formation, and capillary density in skeletal muscle. Male littermate control (L-Control) mice and OXGR1 global knockout (OXGR1-GKO) mice were fed with chow diet at the age of 3−10 weeks. (a) Weekly body weights of male L-Control (*n* = 3) and OXGR1-GKO (*n* = 4) mice. (b) Weekly accumulative food intake of male L-Control (*n* = 3) and OXGR1-GKO (*n* = 4) mice. (c) Body composition of 10 weeks male L-Control (*n* = 7) and OXGR1-GKO (*n* = 12) mice. (d) Running distance of the L-Control (*n* = 8) and OXGR1-GKO (*n* = 6) mice. (e) Skeletal muscle index of the L-Control (*n* = 4) and OXGR1-GKO (*n* = 6) mice. (f and g) The laminin immunofluorescent staining and gastrocnemius muscle average area statistical analysis of the L-Control (*n* = 6) and OXGR1-GKO (*n* = 6) mice. (h and i) Representative images and co-staining of laminin (green) and MyHC I or MyHC IIb (red) in gastrocnemius and statistical analysis of the L-Control (*n* = 5) and OXGR1-GKO (*n* = 5) mice. (j and k) Representative images and quantification of capillary (red) and arteriole (merge) immunofluorescent staining in gastrocnemius of the L-Control (*n* = 6) and OXGR1-GKO (*n* = 6) mice. (l) Quantification of mRNA expression of contractility associated genes in gastrocnemius of the L-Control (*n* = 7) and OXGR1-GKO (*n* = 9) mice. (m) Quantification of mRNA expression of angiogenesis-associated genes in gastrocnemius of the L-Control (*n* = 5) and OXGR1-GKO (*n* = 5) mice. Scale bar in (f, h, and j) represents 50 μm. In (f), (h), and (j), full-field images were retained for each sample. A sample mean from 5 or 6 fields of statistical view, one of which was selected for display. Data information: results are presented as mean ± SEM. In (a) and (b), differences between groups were analyzed for statistical significance by two-way ANOVA followed by *post hoc* Bonferroni tests; In (d), (e), (g), (i), and (k), differences between groups were analyzed for statistical significance by Student’s unpaired *t* test; In (l) and (m), differences between groups were analyzed for statistical significance by multiple comparisons using the Šídák-Bonferroni; *P < 0.05, **P < 0.01.

We generated a global OXGR1 knockout (OXGR1-GKO) mouse model to investigate the requirement of OXGR1 in skeletal muscle function and growth (Supplementary Fig. S1a−d). We found no difference in body weight, food intake, and body composition in OXGR1-GKO mice compared with the littermate control (L-Control) mice (Fig. 2a−c), but the running distance was decreased significantly (Fig. 2d). Furthermore, our findings indicated that the weight, volume, and muscle fiber cross-sectional area in the soleus were significantly decreased in OXGR1-GKO mice (Fig. 2e and Supplementary Fig. S2a−c), whereas there was no change in the gastrocnemius muscle weight and average area (Fig. 2f and g). Based on the findings, the loss of OXGR1 is found to cause attenuation of slow muscles and the impairment of exercise capacity. We performed immunofluorescence staining on oxidized red muscle fiber (MyHC I), glycolytic white muscle fiber (MyHC IIb), capillaries (CD31), and arterioles (α-SMA/CD31) to further explore the role of OXGR1 in skeletal muscle metabolism. The results showed that the proportion of type I fibers in gastrocnemius (Fig. 2h and i) and the capillary density in gastrocnemius and extensor digitorum longus (Fig. 2j and k) were decreased dramatically in OXGR1-GKO mice than the L-Control mice. Congruously, the succinate dehydrogenase (SDH) positive fibers and the expression of MyHC I protein in gastrocnemius were decreased, and the expression of MyHC IIb protein in gastrocnemius were increased (Supplementary Fig. S3a–d, f and h). However, there was no difference in the expression of MyHC IIa and MyHC IIx in gastrocnemius (Supplementary Fig. S3c and e–g), proportion of type I/IIb fibers in extensor digitorum longus and soleus (Supplementary Fig. S2d–f), and vascular density in soleus (Supplementary Fig. S2g–i) in OXGR1-GKO mice when compared with the L-Control mice. Particularly, the mRNA levels of contractile markers (MYH11) (Fig. 2l) were decreased, but the mRNA levels of angiogenesis markers (CD31 and VEGFR2) (Fig. 2m) were not different in gastrocnemius in OXGR1-GKO mice. This suggested that the loss of OXGR1 results in a reduction of oxidized red muscle fibers, and a suppression of contractile marker mRNA expression.

### AKG promotes the transformation of slow fiber and vascular density in skeletal muscle

**Figure 3:**
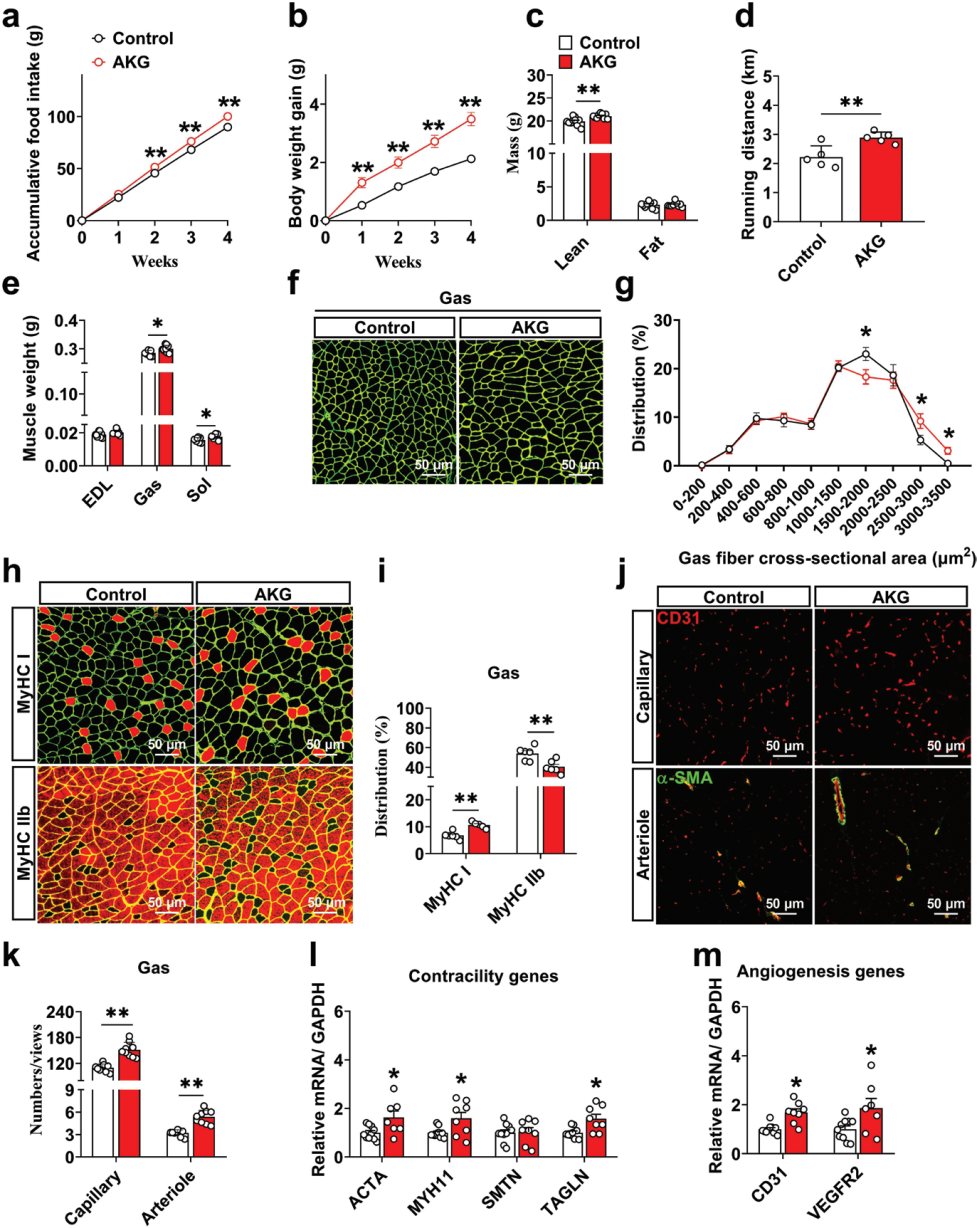
Dietary supplementation of AKG promotes skeletal muscle function and angiogenesis. C57BL/6J male mice were fed with chow diet supplemented 2% AKG-add water for 4 weeks at the age of 8 weeks. (a) Weekly body weights of male control (*n* = 10) and AKG (*n* = 10) groups of mice. (b) Weekly accumulative food intake of male control (*n* = 10) and AKG (*n* = 10) groups of mice. (c) Body composition of 12-week-old control (*n* = 10) and AKG (*n* = 9) groups of mice. (d) Running distance of control (*n* = 5) and AKG (*n* = 5) groups of mice. (e) Skeletal muscle index of control (*n* = 9) and AKG (*n* = 9) groups of mice. (f and g) The laminin immunofluorescent staining and gastrocnemius muscle average area statistical analysis of control (*n* = 7) and AKG (*n* = 6) groups of mice. (h and i) Representative images and co-staining of laminin (green) and MyHC I or MyHC IIb (red) in gastrocnemius and statistical analysis of control (*n* = 6) and AKG (*n* = 7) groups of mice. (j and k) Representative images and quantification of capillary (red) and arteriole (merge) immunofluorescent staining in gastrocnemius of control (*n* = 9) and AKG (*n* = 9) groups of mice. (l) Quantification of mRNA expression of contractility associated genes in gastrocnemius of control (*n* = 10) and AKG (*n* = 8) groups of mice. (m) Quantification of mRNA expression of angiogenesis associated genes in gastrocnemius of control (*n* = 10) and AKG (*n* = 8) groups of mice. Scale bar in (f), (h), and (j) represents 50 μm. In (f), (h), and (j), full-field images were retained for each sample. A sample mean from 5 or 6 fields of statistical view, one of which was selected for display. Data information: results are presented as mean ± SEM. In (a) and (b), differences between groups were analyzed for statistical significance by two-way ANOVA followed by *post hoc* Bonferroni tests; In (d), (e), (g), (i), and (k), differences between groups were analyzed for statistical significance by Student’s unpaired *t* test; In (l) and (m), differences between groups were analyzed for statistical significance by multiple comparisons using the Šídák-Bonferroni; *P < 0.05, **P < 0.01.

To further discover the functions of OXGR1, we fed mice with AKG-add water, an endogenous agonist of OXGR1, and observed the growth performance. In accordance with Yuan *et al*. [6], our results also showed that AKG significantly increased food intake, body weight gain, and lean mass (Fig. 3a–c). Moreover, the running distance (Fig. 3d), skeletal muscle weight (Fig. 3e), and skeletal muscle fiber average area (Fig. 3f and g, and Supplementary Fig. S4a–c) were increased by AKG. Surprisingly, the proportion of type I muscle fibers in the gastrocnemius and extensor digitorum longus was evidently increased by AKG, whereas the proportion of type IIb fibers in the gastrocnemius and soleus was dramatically decreased under the same treatment (Fig. 3h and i, and Supplementary Fig. S4d–f). Coincidentally, the SDH positive fibers and the expression of MyHC I were increased (Supplementary Fig. S5a–d), and the expression of MyHC IIb were decreased (Supplementary Fig. S5j and k). Furthermore, AKG significantly increased the density of arteriole and capillary in skeletal muscle (Fig. 3j and k, Supplementary Fig. S4g–i), as well as the mRNA levels of contractile markers (ACTA, MYH11, and TAGLN) (Fig. 3l) and angiogenesis markers (CD31 and VEGFR2) (Fig. 3m). Based on these results, it is suggested that AKG promotes skeletal muscle hypertrophy and oxidative fiber transformation, and elevates vascular density.

### AKG increases vascular perfusion in skeletal muscle

**Figure 4:**
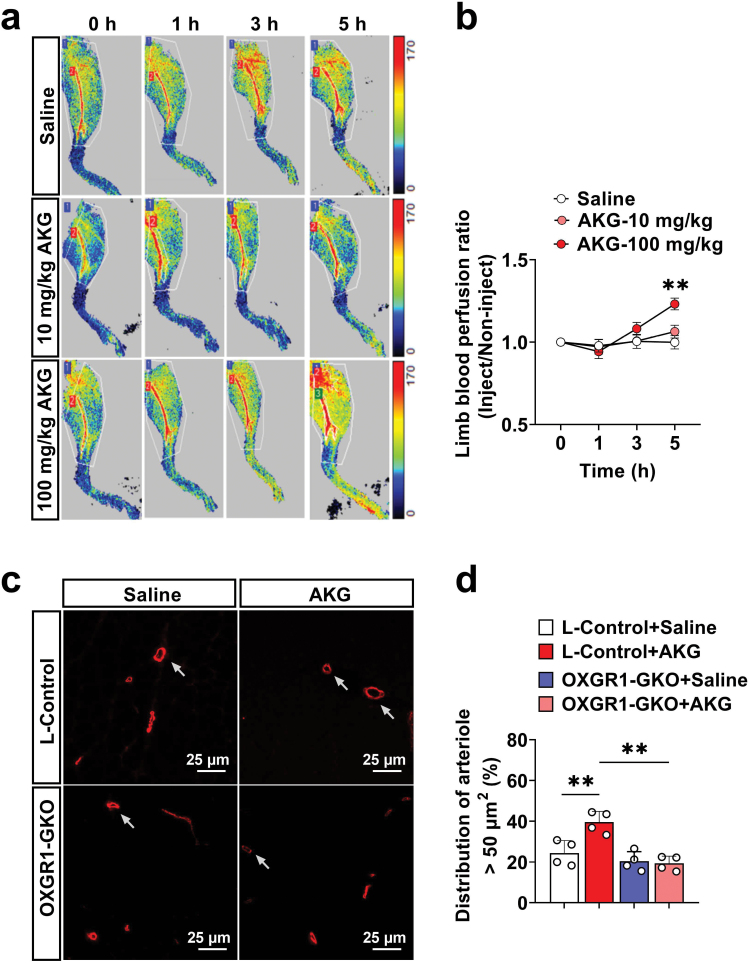
Acute AKG administration increases arteriole diastole and blood flow of lower limbs in mice by OXGR1. (a, b) Representative laser speckle perfusion images and quantification of blood perfusion in lower limbs of mice at 1, 3, and 5 h after being injected with physiological saline (*n* = 7), 10 mg/kg (*n* = 6) and 100 mg/kg AKG (*n* = 8). The blood flow ratio of each sample was compared with the blood flow basal value before self-injection (0 h). (c, d) Representative arteriole-*α*-SMA-positive (red) density images and quantitative arteriole >50 μm in gastrocnemius of L-Control and OXGR1-GKO mice at 5 h after being injected physiological saline and 100 mg/kg AKG (*n* = 4). The scale bar in (c) represents 25 μm. In (c), a sample mean from 3 fields of statistical view, one of which was selected for display. Data information: results are presented as mean ± SEM. In (b), differences between groups were analyzed for statistical significance by two-way ANOVA followed by *post hoc* Bonferroni tests. In (d), differences between groups were analyzed for statistical significance by one-way ANOVA followed by *post hoc* Dunnett’s tests, **P < 0.01.

We used laser speckle to test the blood flow ratio of the lower extremities after acute injection of 10 and 100 mg/kg AKG, compared with the saline injection to determine the vascular perfusion effect of AKG in skeletal muscle. Accordingly, we found a significant increase in blood perfusion of the lower limbs 5 h after injection of 100 mg/kg AKG (Fig. 4a and b). Subsequently, we found that AKG relaxed the VSM cells (VSMCs) depending on time and dose (Supplementary Fig. S8a and b). Furthermore, we measured the vascular caliber after AKG acute injection in gastrocnemius, and found that the proportion of vascular with a diameter larger than 50 µm was significantly increased, but it was ineffective in OXGR1-GKO mice (Fig. 4c and d). We acutely injected AKG into adrenalectomized mice to rule out the effect of epinephrine on muscle perfusion and found that blood perfusion of the lower limb was still increased (Supplementary Fig. S6a and b). Based on these results, AKG can increase vascular perfusion at the skeletal muscle level in an adrenaline-independent manner.

### AKG promotes the transformation of slow fiber and vascular density in skeletal muscle by OXGR1

**Figure 5:**
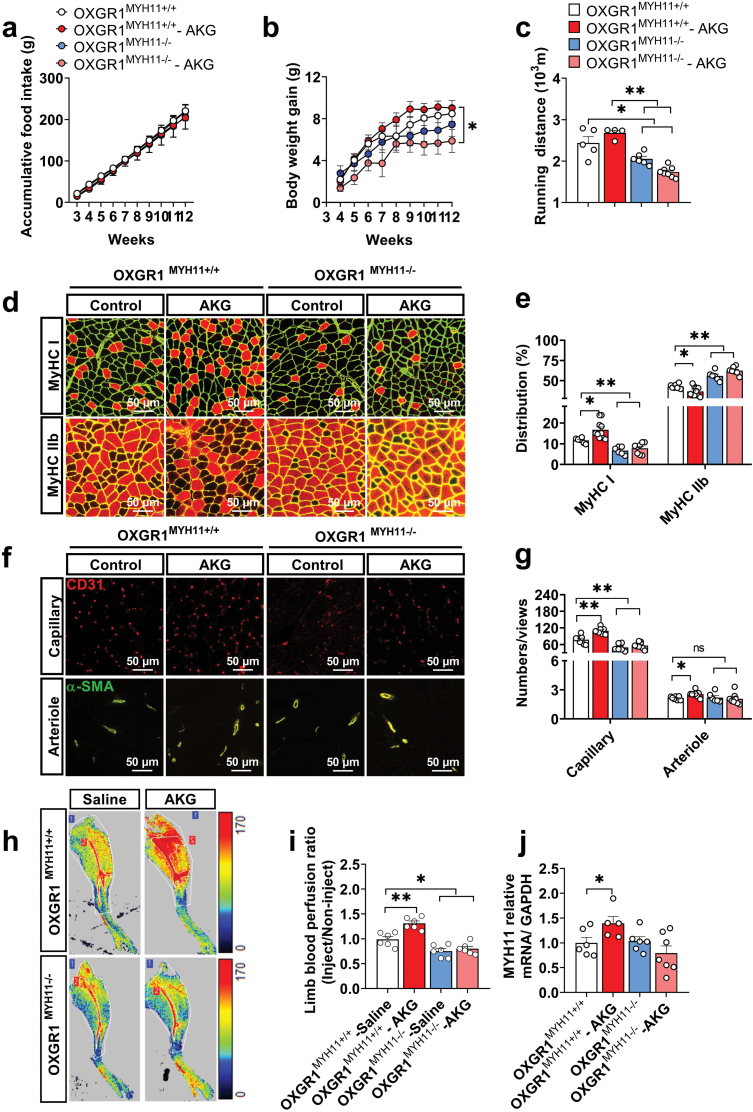
OXGR1 in vascular smooth muscle mediated the effects of AKG on skeletal muscle function and angiogenesis. OXGR1^MYH11+/+^ or OXGR1^MYH11−/−^ female mice were fed with chow diet supplemented with 2% AKG-add water for 9 weeks at the age of 3 weeks. (a) Weekly body weights of OXGR1^MYH11+/+^ (*n* = 7), OXGR1^MYH11+/+^ with AKG (*n* = 10), OXGR1^MYH11−/−^ (*n* = 6), and OXGR1^MYH11−/−^ with AKG (*n* = 8) groups of mice. (b) Weekly accumulative food intake of OXGR1^MYH11+/+^ (*n* = 7), OXGR1^MYH11+/+^ with AKG (*n* = 10), OXGR1^MYH11−/−^ (*n* = 6), and OXGR1^MYH11−/−^ with AKG (*n* = 8) groups of mice. (c) Running distance of 12-week-old OXGR1^MYH11+/+^ (*n* = 6), OXGR1^MYH11+/+^ with AKG (*n* = 4), OXGR1^MYH11−/−^ (*n* = 6), and OXGR1^MYH11−/−^ with AKG (*n* = 8) groups of mice. (d and e) Representative images and co-staining of laminin (green) and MyHC I or MyHC IIb (red) in gastrocnemius and statistical analysis of 12-week-old OXGR1^MYH11+/+^ (*n* = 6), OXGR1^MYH11+/+^with AKG (*n* = 10), OXGR1^MYH11-/-^ (*n* = 6), and OXGR1^MYH11+/+^with AKG (*n* = 7) groups of mice. (f and g) Representative images and quantification of capillary (red) and arteriole (merge) immunofluorescent staining in gastrocnemius of OXGR1^MYH11+/+^ (*n* = 7), OXGR1^MYH11+/+^ with AKG (*n* = 8), OXGR1^MYH11−/−^ (*n* = 6), and OXGR1^MYH11−/−^ with AKG (*n* = 8) groups of mice. (h and i) Representative laser speckle perfusion images and quantification of blood perfusion in lower limbs of OXGR1^MYH11+/+^ and OXGR1^MYH11−/−^mice at 5 h after being injected with physiological saline or 100 mg/kg AKG (*n* = 6). The blood flow ratio of each sample was compared with the blood flow basal value before self-injection (0 h). (j) Quantification of mRNA expression of MYH11 in gastrocnemius of OXGR1^MYH11+/+^ (*n* = 6), OXGR1^MYH11+/+^ with AKG (*n* = 5), OXGR1^MYH11−/−^ (*n* = 6), and OXGR1^MYH11+/+^ with AKG (*n* = 7) groups of mice. Scale bar in (d) and (f) represents 50 μm. In (d) and (f), full-field images were retained for each sample. A sample meaned from 5 or 6 fields of statistical view, one of which was selected for display. Data information: results are presented as mean ± SEM. In (a and b), differences between groups were analyzed for statistical significance by two-way ANOVA followed by *post hoc* Bonferroni tests. In (c), (e), (g), (i), and (j), differences between groups were analyzed for statistical significance by one-way ANOVA followed by *post hoc* Tukey’s tests, *P < 0.05, **P < 0.01.

Our study used the MYH11-Cre × OXGR1-FloxP system to generate OXGR1 specific knockdown mice in order to investigate the role of OXGR1 in the effects of AKG on skeletal muscle metabolism (Supplementary Fig. S7a−e). OXGR1^MYH11+/+^ or OXGR1^MYH11−/−^ female mice were fed with 2% AKG-add water for 9 weeks. There was no difference in food intake, but the weight gain of the knockdown mice with AKG body was decreased compared with control mice with AKG (Fig. 5a and b), and the running distances of the knockdown mice were significantly decreased compared with the control mice (Fig. 5c). In the knockdown mice, the proportion of type I fibers in gastrocnemius was dramatically decreased compared with the control mice. Meanwhile, AKG improved the proportion of type I fibers in gastrocnemius in control mice, but it was ineffective in the knockdown mice (Fig. 5d and e). Inversely, the proportion of type IIb fibers in gastrocnemius was significantly increased in the knockdown mice. At the same time, AKG decreased the proportion of type IIb fibers in gastrocnemius in the control mice, but it was ineffective in the knockdown mice (Fig. 5d and e). Besides, we found that capillary density in gastrocnemius was decreased in the knockdown mice compared with the control mice. Similarly, AKG increased arteriole and capillary density, but it was ineffective in the knockdown mice (Fig. 5f and g), and mRNA levels of MYH11 were also the case (Fig. 5j). According to these results, AKG promotes the transformation of oxidative red muscle and increases the vascular density mediated by OXGR1. To investigate whether AKG increases vascular perfusion in skeletal muscle through OXGR1, we tested the blood flow ratio of the lower extremities and found that AKG expedited blood flow in the lower limbs (Fig. 5h and i), but it was ineffective in the knockdown mice. This outcome indicated that OXGR1 mediates AKG increasing vascular perfusion in skeletal muscle.

### OXGR1 is required for AKG relaxation of VSMCs

**Figure 6:**
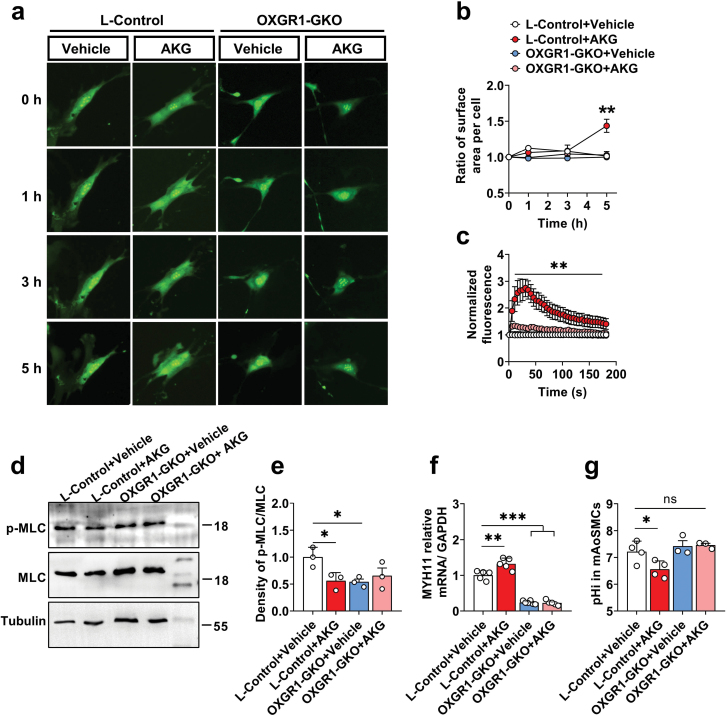
OXGR1 mediates AKG-induced relaxation of vascular smooth muscle cells. (a and b) Representative images and quantification of ratio of surface area treated with vehicle or 100 μmol/L AKG in primary AoSMCs from the L-Control and OXGR1-GKO mice (*n* = 8). The surface area ratio of each sample was compared with the surface area value before treatment (0 h). (c) Intracellular calcium ion changes treated with vehicle or 100 μmol/L AKG in primary AoSMCs from the L-Control and OXGR1-GKO mice (*n* = 6). The fluorescence ratio of each sample was compared with the fluorescence value before treatment (0 s). (d and e) Immunoblots and quantification of p-MLC/MLC protein treated with vehicle or 100 μmol/L AKG in primary AoSMCs from the L-Control and OXGR1-GKO mice (*n* = 3). (f) Quantification of mRNA expression of MYH11 genes treated with vehicle or 100 μmol/L AKG in primary AoSMCs from the L-Control or OXGR1-GKO mice (*n* = 5). (g) Intracellular pH treated with vehicle or 100 μmol/L AKG in primary AoSMCs from L-Control and OXGR1-GKO mice (*n* = 4). Data information: results are presented as mean ± SEM. In (b) and (c), differences between groups were analyzed for statistical significance by two-way ANOVA followed by *post hoc* Bonferroni tests, **P < 0.01. In (e), (f), and (g), differences between groups were analyzed for statistical significance by one-way ANOVA followed by *post hoc* Dunnett’s tests, *P < 0.05, **P < 0.01, ***P < 0.001.

To examine whether OXGR1 facilitates the relaxation effect of AKG at the cellular level, we extracted the primary aortic smooth muscle cells (AoSMCs) from the L-Control mice and OXGR1-GKO mice and treated them with AKG. After incubating with 100 μmol/L AKG for 5 h, the cell proportion was found to be dramatically increased, and it was blocked in the OXGR1-GKO group (Fig. 6a and b). Additionally, we found that AKG increased instantaneous calcium ion concentration and decreased cellular pH in the L-Control group, both of which were blocked in the OXGR1-GKO group (Fig. 6c and g), and Ca^2+^ homeostasis and intracellular acid–base balance involved in the relaxation effect of AKG (Supplementary Fig. S9a−h). We also discovered that AKG decreased the phosphorylation level of myosin light chain (MLC) and increases mRNA levels of MYH11, both of which were blocked in the OXGR1-GKO group (Fig. 6d−f). It is worth noting that mRNA levels of MYH11 were significantly decreased in the OXGR1-GKO group compared with the L-Control group (Fig. 6f). These findings illustrated that the OXGR1 mediates relaxing effect of smooth muscle cells by AKG.

## Discussion

Skeletal muscle plays a vital role in several functions of the human body. From a metabolic point of view, the role of skeletal muscle comprises promoting basic energy metabolism, storing important substrates, including amino acids and carbohydrates, generating heat to maintain core temperature, and consuming most of the oxygen and fuel during physical activity and exercise [11]. In skeletal muscle, there are a variety of cell types with the complex adaptive response and expressing G protein conjugate receptors [12]. Thus, studying the G protein receptors in these cells can provide a better understanding of the process of metabolic coordination in various cell types, which could facilitate the treatment of skeletal muscle diseases. GPR99 was primarily described as OXGR1. Research has indicated that OXGR1 regulates the growth of axons, and the position of neurons is closely associated with blood vessels due to the similar molecular and signaling mechanisms between axon guidance, neuronal migration, and vascular guidance and growth [13]. Although prior studies reported a low expression of OXGR1 in skeletal muscle, we systematically examined the expression of OXGR1 in skeletal muscle and found that the function of skeletal muscle was weakened in the OXGR1-GKO mice. According to immunofluorescence staining, OXGR1 is found to be located in VSMCs of skeletal muscles, suggesting its role in systolic and diastolic functions of arteries in skeletal muscle.

The function of the receptor is associated with its distribution, and the literature reports that OXGR1 is primarily distributed in the kidneys, smooth muscles, and testis [9], which is a crucial receptor for regulating the acid–base balance in the kidneys [14]. Shirasaki *et al*. reported that OXGR1 is distributed in VSMs of the nasal mucosa [15]. Bankova *et al*. proposed that OXGR1 is a high-affinity leukotriene E4 (LTE4) receptor, a stable cystic weak agonist that shrinks the smooth muscles of the airways but causes airflow obstruction and inflammation of the lungs in asthma patients [10]. In line with this, our results demonstrated the distribution of OXGR1 in VSM. The primary function of VSM is controlling vascular vasomotion. Studies have suggested that the coupling of OXGR1 receptors with Gq protein may lead to vascular constriction, elevated blood pressure, hypertension formation, etc. [16], but no specific results have been reported. Blood flow to skeletal muscles is closely associated with metabolites, and there was a view that contracted muscles release active substances that result in vascular diastolic [4]. There have been studies of local metabolites, including nitric oxide, adenosine triphosphate, arginine, and lysine, that have a diastolic effect on blood vessels [4, 5], but they lacked clear evidence. OXGR1 functions as a receptor for the TCA cycle intermediate AKG. A variety of metabolites may regulate it as the distribution of OXGR1 is universal and is regulated by AKG metabolites and analogs in the form of local secretion [14]. It was concluded from these results that OXGR1 is closely related to vascular function in skeletal muscle.

The steady state of the skeletal muscle is a dynamic balance of chemical and mechanical factors, in which the microvascular system plays an important role in maintaining health and muscle function [17]. In order to promote muscle fiber growth following exercise, proper muscle fiber perfusion is essential for the delivery of oxygen, nutrients, and growth factors. Snijders *et al*. had demonstrated that a lower density of muscle fiber capillaries in older adults results in a slow response to recovery after exercise training [18]. In particular, we found that skeletal muscle capillary density is attenuated, and oxidative red fibers are reduced on the OXGR1-GKO model. The arteries and veins in the skeletal muscles form complex networks whose density varies according to the type of muscle. The arteries are further divided into smaller networks of arteries, arranged perpendicular to the muscle fiber axis, and eventually branched into capillaries. This network is connected to small veins and veins, forming a complex vascular mesh structure containing bundles of muscle fibers. VSMCs, endothelial cells, and peripheral cells are the major cell types of blood vessel walls. Since different muscle types need different amounts of energy, the density of blood vessels and the thickness of capillaries are also different in order to meet their oxygen needs. Yuan *et al*. found that OXGR1 is located in the adrenal glands [6], a neuroendocrine converter that converts nerve information into hormone information. Adrenaline myelin secretes epinephrine and norepinephrine; α-adrenaline can activate the pathway that affects arterial contraction and the perfusion of blood flow to the lower extremities [19]; β-adrenaline can activate the pathway that induces muscle hypertrophy [20]. To eliminate these factors, we used the Cre-FloxP system to specifically knock out OXGR1 in VSMs, but the results showed that skeletal muscle capillary density is attenuated, oxidative red fibers are reduced, and endurance exercise performance is damaged. Considering the loss of OXGR1, only the soleus muscles atrophy, we speculate that the soleus muscle capillary density is rich, which is greatly affected, whereas other muscles have a compensation effect. These data suggested that the absence of OXGR1 will damage vascular perfusion, leading to a reduction in the surface area of microvascular exchange, oxygen, nutrients, and hormones, thereby impairing the health and function of skeletal muscle.

AKG, a natural ligand of OXGR1, is a myometabolite responsible for many beneficial metabolic effects during exercise. While previous studies had demonstrated that AKG can promote muscle hypertrophy [6], our data indicated that AKG not only promotes skeletal muscle hypertrophy, but also enhances exercise performance, the proportion of type I fibers, and vessels density in skeletal muscles. In this study, we examined the phenotype of AKG in skeletal muscle dependent on OXGR1. Notably, the role of AKG in promoting skeletal muscle hypertrophy is not controlled by OXGR1, but the endurance exercise performance, the proportion of slow muscle fibers, and vessels density are controlled by OXGR1. It is notable that shear stress is a crucial factor in new capillaries [21, 22]. Blood flow in the muscle microvascular influences capillary density [23], and is determined by vascular diastole [24]. Furthermore, the contraction and diastole of the blood vessel smooth muscles are mostly facilitated by G protein conjugate receptors [25]. We found that OXGR1 mediates the effect of AKG promoting blood flow in the lower extremities, but it is unclear whether the absence of OXGR1 would result in loss of vascular perfusion, which could be explained by the balance loss of OXGR1’s involvement in the diastolic effect [26]. After acute injection in the OXGR1^−/−^ mice, blood flow recovers and decreases, indicating AKG and OXGR1 have independent and coordinated effects. Interestingly, AKG has been shown to extend the lifespan of mice [27], and studies have found that capillary density in skeletal muscles is lost with aging, along with a decrease in muscle mass and strength [28]. However, we found a decrease in OXGR1 expression in older mice (unpublished). These results suggested that the AKG/OXGR1 signaling pathway may play a pivotal role in reducing muscle aging, by regulating the formation of slow muscle fibers and the vascular density in the skeletal muscle.

Vascular blood flow is determined by vasomotion, which is regulated by the conduction and integration of intercellular vascular activity signals [29]. Vasoconstriction depends on the contraction of VSMCs, triggered by free calcium ions in the cytoplasm. However, the change of intracellular calcium ion concentration is instantaneous, and the contraction reaction process requires long-term inhibition of myosin phosphatase to cause phosphorylation of MLC [30]. Intracellular pH (pH_i_) is a physiological parameter closely associated with the contraction of VSM. In arteries, resting tension appears to be tightly coupled with pH_i_, vascular contraction is mediated by intracellular alkalinization, and vascular dilation is triggered by intracellular acidification [31]. Protons passing through the VSMC membrane are not in electrochemical equilibrium, thus they are constantly exchanged at voltage and ion concentration gradients until equilibrium is reached, and are mainly transported through sodium ions, hydrogen ion exchangers, and sodium conjugated bicarbonate transport carriers [32]. Vascular tension is related to the concentration gradient of sodium ions [33], and studies have found that the AKG/OXGR1 signaling pathway contributes to maintaining the NaCl steady state and the ability to maintain acid–base balance in the OXGR1^−/−^ mice has significantly attenuated [14]. We found that AKG can induce a lower pH in VSMCs, which is abolished in the OXGR1-GKO VSMCs. We investigated the contraction marker protein and gene expression in order to support the idea that the AKG/OXGR1 signaling pathway is involved in the relaxation of VSM. The results showed that phosphorylation of MLC decreased and MYH11 transcription increased. Besides, changes in transient calcium ions in cells significantly increased after AKG addition. It is generally understood that the relaxation and contraction of VSMCs are regulated by the concentration of intracellular calcium ions, but in fact, they are dependent on calcium sensitivity. The above results just proved that the calcium sensitivity being passivated at a low pH value would remain of the result of relaxation in VSMC [34]. It has been confirmed that calcium signaling and intracellular acid–base balance are key signals and components for AKG/OXGR1 to regulate relaxation, but the pathway of OXGR1 in the diastolic state of the ion mechanism requires further study. Importantly, AKG does not affect vascular endothelial cell proliferation [35], indicating that AKG may only affect relaxation for VSMCs. The serum AKG levels were elevated by about 90−120 µmol/L after exercise in mice [6], constricted skeletal muscles have a strong vascular diastolic capacity that increases blood flow by approximately a hundred times in dynamic motion, and motor vasodilation comes from the balance between vascular contraction and vascular relaxation signals [36]. Vasodilation induced by AKG/OXGR1 can also be interpreted as part of exercise congestion.

**Figure 7:**
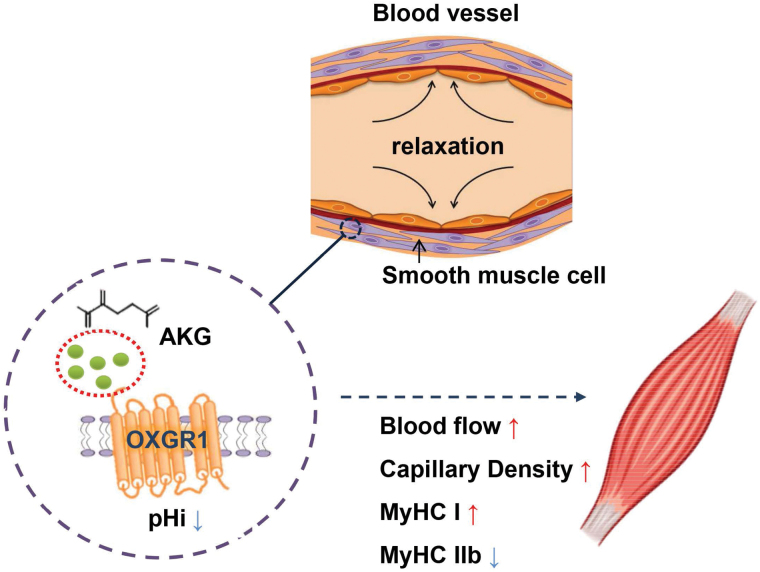
OXGR1 of vascular smooth muscle in skeletal muscle mediated AKG promoting blood flow and metabolism. AKG decreased the intracellular pH in vascular smooth muscle through OXGR1, resulting in vasodilation of skeletal muscle. OXGR1 mediates AKG to promote blood flow and capillary density, which influences the distribution of MYHC I fiber types and metabolism in skeletal muscle.

In conclusion, the results indicated that OXGR1 is one of the critical receptors of gastrocnemius capillary vascularization and blood supply, which influences the metabolism and fiber type transition of skeletal muscle, and mediates the effects of AKG on relaxing smooth muscle cells (Fig. 7) [37, 38]. Moreover, this study explained the theory of exercise-induced muscle fiber vascularization, provided a dietary strategy basis for AKG addition in animal feed and safety in humans, and created a new treatment for AKG/OXGR1 signaling pathway in skeletal muscle aging and cardiovascular disease.

## Materials and methods

### Animals

Mice (C57BL/6 mice were purchased from the Animal Experiment Center of Guangdong Province Guangzhou, Guangdong, China) were housed in a temperature-/humidity-controlled environment (24°C ± 2°C/70% ± 10%) in a 12-h light/12-h dark cycle (6 a.m./6 p.m.). Unless otherwise stated, the mice were maintained *ad libitum* on standard mouse chow (Protein 18.0%, Fat 4.5%, and Carbohydrate 58%; Guangdong Medical Science Experiment Center, Guangzhou, Guangdong, China) and water. All groups within one experiment contain individual mice with the same strain and sex, showing similar body weight and age. All used mice aged between 10 and 20 weeks when they were sacrificed. To study the metabolic effects of AKG, C57BL/6 mice were used in acute or short-term experiments. The OXGR1-GKO mice (Shanghai Research Center for Model Organisms, Shanghai, China) and the OXGR1-FloxP mice (Beijing Viewsolid biotech Limited company, Beijing, China) were generated on a C57BL/6 background. They were used to study the metabolic effects of short-term AKG supplementation. Care of all animals and procedures in South China Agricultural University conformed to “The Instructive Notions concerning Caring for Laboratory Animals” issued by the Ministry of Science and Technology of the People’s Republic of China and were approved by the Animal Subjects Committee of South China Agricultural University.

### OXGR1-GKO mouse model

OXGR1-GKO mouse model was generated by Shanghai Model Organisms Center, Inc. The guide RNAs targeting exon 4 of OXGR1 gene were designed using CRISPR/Cas9 strategy as shown in a specific scheme. The Cas9 mRNA was *in vitro* transcribed using mMESSAGE mMACHINE T7 Ultra Kit (Ambion, TX, USA), according to the manufacturer’s instructions. Two sgRNAs were designed to delete the OXGR1 protein-coding region using the online guide design tools provided by Feng Zhang lab. The target sequences of two sgRNAs were 5ʹ-GTTTAACCTCTAACTTCCAC-3ʹ and 5ʹ-TTAAAGGCTCGAAGGCTAAC-3ʹ. The sgRNAs were *in vitro* transcribed using the MEGAshortscript Kit (Thermo Fisher, USA), and subsequently purified using MEGAclear™ Kit (Ambion, Life Technologies, USA). The mixture of Cas9 mRNA and sgRNAs were co-injected into the zygotes of C57BL/6 mouse by microinjection. F0 mice were genotyped using the primer pairs (forward: 5ʹ-TATACCAGCTGTTTTTCTTGTTGC-3ʹ; reverse: 5ʹ-GATGCGTGGCTGTTTATGTCA-3ʹ). The genotype of positive F0 was confirmed by sequencing. The positive F0 mice were chosen and crossed with C57BL/6 mice to produce F1 mice. The genotype of F1 mice was identified by PCR and confirmed by sequencing. The mRNA expression of OXGR1 was also compared between the L-Control mice and OXGR1-GKO mice. F1 mice with protein-coding region deletion in exon 4 were used to intercross to obtain the homozygous OXGR1-GKO mice [6].

### OXGR1 VSM-specific knockdown model

The OXGR1-FloxP mouse was generated in Beijing Viewsolid Biotech Co. Ltd (Beijing, China). The DNA of the intron target gene was cut by CRISPR technology. The mouse was provided to insert Floxp at both ends of a specific exon through homologous recombination repair of DNA (loxp1-forward: 5ʹ-gccagaggattcagaatggtatc-3ʹ; loxp1-reverse: 5ʹ-ctgtcggtttatggtgagttc-3ʹ; loxp2-forward: 5ʹ-cttaaaggctcggaaggcta-3ʹ; loxp2-reverse: 5ʹ-gtgaatgcgtggctgtt-3ʹ). The smMHC/Cre/eGFP (MYH11-Cre,-EGFP, 007742, The Jackson Laboratory) transgene was designed by placing a Cre recombinase gene, internal ribosomal entry site, and an enhanced green fluorescent protein (EGFP) gene all downstream of the 16 kb mouse smooth muscle myosin heavy chain (smMHC or MYH11) promoter fragment (transgene-forward: 5ʹ-GCGGTCTGGCAGTAAAA ACTATC-3ʹ; transgene-reverse: 5ʹ-GTGAAACAGCATTGCTGTCACTT-3ʹ; internal positive control-forward: 5ʹ-CTAGGCCACAGAATTGAAAGATCT-3ʹ; internal positive control-reverse: 5ʹ-GTAGGTGGAAATTCTAGCATCATCC-3ʹ). The smooth muscle myosin heavy chain (smMHC or MYH11) promoter directed bicistronic Cre and EGFP protein expression to smooth muscle cells during development as well as in the adult mouse.

The Cre recombinase gene was specifically expressed in VSM of the OXGR1-Floxp mice, so that the specific exon of OXGR1 was deleted, OXGR1 did not translate or undergo frameshift mutation, and the OXGR1 protein was inactivated. The mice (F2) were identified as the knockout group, which were inserted with OXGR1-LoxP1, OXGR1-LoxP2 and MYH11-Cre. The mice (F2) without MYH11-Cre were used as the control group, while were inserted with LoxP1 and LoxP2. The F2 mice were produced by crossing between F1 identified heterozygous male mice and the inserted at sites LoxP1 and LoxP2 female mice (F0). The F1 mice were produced by crossing between the inserted at sites LoxP1 and LoxP2 female mice (F0) and the transferred MYH11-Cre male mice (F0).

Food intake and body weight of the mice were recorded from the third week after drinking water supplemented with 2% AKG.

### Adrenalectomized mice

According to the reference from Yuan *et al*. [6], 12-week-old C57BL/6 mice were anesthetized by inhaling isoflurane, followed by receiving bilateral adrenalectomy or sham surgery. The mice were then rehabilitated in an environment (24°C ± 2°C/70% ± 10%) for 2 weeks, after which the mice will be used in followed experiments.

### Body composition

At the end of the experiment, body composition was determined using a nuclear magnetic resonance system (Body Composition Analyzer MiniQMR23-060H-I; Niumag Corporation, Shanghai, China).

### Running endurance test

First, the mice were warmed up at a speed of 8 m/min for 5 min on the FT-200 Animal treadmill at 0°C. The mice would rest for 5 min, and then the running endurance test was performed at an initial speed of 11.2 m/min, followed by an increase of speed at 3−4 m/min every subsequent 5 min. Exhaustion of the mice was defined as the inability of the mice back to run within 10 s after direct contact on an electric grid despite mechanical prodding. Running distance was measured until exhaustion. The above tests refer to the previous study [39].

### Laser speckle test

12-week-old C57BL/6 mice were anesthetized by inhaling isoflurane, followed by using depilatory cream to dehair the lower limbs and placing the mice on a heating pad to keep at a constant temperature of 37°C. The blood flow value of lower limbs was recorded at different time points after an acute injection of saline or 100 mg/kg AKG using the blood flow imaging system (LSCI-S1, Shanghai Defen Biotechnology Co., Ltd.), followed by taking the averaged blood flow value in 1 min at each time point and calculating the ratio relative to the initial time point. After recording, the lower limb muscles were collected, followed by freezing in liquid nitrogen immediately to maintain the muscle morphology, and then the muscles were stored at −80°C.

### Immunofluorescence staining

For staining of muscle sections, the frozen sections at −80°C were taken to warm back for 5 min, sealed at room temperature for 10 min. Subsequently, the cocktail containing the primary antibodies (OXGR1 antibody: ab140630, 1:1000, Abcam; α-SMA antibody: sc-56499, 1:1000, Santa Cruz; Laminin antibody: L9393, 1:2000, sigma; CD31 (PECAM-1) antibody: DIA-310, 1:1000, Dianova; MYHC I antibody: BA-D5, 1:200, DSHB; MYHC IIb antibody: BF-F3, 1:200, DSHB) and blocking buffer (10 mL blocking buffer was prepared with PBS containing 0.5 ml goat serum, 0.2 g BSA, 0.2 mL 10% Triton X-100, and 0.01 g sodium azide) were incubated on the surface of muscle sections in a wet box at 4°C overnight. Subsequently, the muscle sections were washed with PBS three times (5 min each), followed by incubating with the secondary antibodies (Goat anti-mouse IgG H+L Cy3: JAC-115-005-003, 1:2000, Jackson; Goat anti-rabbit IgG H+L 488: JAC-111-005-003, 1:2000, Jackson; Goat anti-rat IgG H+L Cy3: JAC-112-005-003, 1:2000, Jackson) in PBS (1:2000) at room temperature. The muscle sections were sealed after incubating Sudan Black for 5 min and washed with PBS three times (5 min each), and were observed under the fluorescence microscope (Nikon Instruments, Tokyo, Japan).

### Western blot analysis

Cells or muscles were cracked by the RIPA lysis buffer containing 1 mmol/L PMSF. For the nuclear or cytoplasmic protein extraction, proteins were isolated according to the procedure of the nuclear extraction kit (Solarbio, SN0020). Protein concentration was determined using a BCA protein assay kit. After sodium dodecyl sulfate polyacrylamide gel electrophoresis gels, total protein lysates (20 μg) were immunoblotted with primary antibody (OXGR1 antibody: ab140630, Abcam; MYHC I antibody: BA-D5, 1:500, DSHB; MYHC IIa antibody:SC-71, 1:500, DSHB; MYHC IIx antibody: BF-35, 1:500, DSHB; MYHC IIb antibody: BF-F3, 1:500, DSHB; β-Actin antibody: AP0060, 1:2000, Bioworld; β-Tubulin antibody: AP0064, 1:2000, Bioworld; Phospho-Myosin Light Chain 2 (Ser19) antibody, 3675S, Cell Signaling Technology), followed by incubating with goat anti-rabbit HRP-conjugated secondary antibody (1:50000, bs-0294D or bs-0295G, Bioss, Woburn, MA, USA). The levels of β-Tubulin or β-Actin served as the loading control. Protein expression levels were determined using MetaMorph software ImageJ (National Institutes of Health, USA).

### The SDH staining

SDH staining was performed as previously described [40]. Briefly, muscle sections (7 μm) were incubated in liquid (6 mmol/L CaCl_2_, 0.3% glacial acetic acid, pH 4.4) for 10 min, flushed by Tris-CaCl_2_ eluent buffer (0.1 mol/L Tris, 18 mmol/L CaCl_2_) twice, and then incubated in 37°C pre-heated SDH eluent buffer (0.1 mol/L sodium succinate, 0.18 mmol/L tetranitroblue tetrazolium chloride (NBT), 0.81% N, N-dimethylformamide, 0.23 mol/L Tris, pH = 7.4) for 45 min. The sections were washed with distilled water twice and then incubated in 37°C pre-heated ATPase eluent buffer (3 mmol/L adenosine 50-triphosphate disodium salt, 0.2 mol/L Tris, 18 mmol/L CaCl_2_, 50 mmol/L KCl, pH 9.4) for 30 min The sections were then washed with distilled water twice, incubated in 2% CoCl_2_ for 4 min, and washed carefully with distilled water twice. Sections were then incubated in 2% ammonium sulfide for 30 s, followed by careful washing with distilled water twice. After staining with Ehrlich’s hematoxylin, sections were sent for dehydration in alcohol and fixated by neutral balsam. The sections were observed under the olympus CX41 microscope (Olympus Corporation, Japan).

### RNA extraction, reverse transcript, and qPCR

Total RNA was extracted from smooth muscle cells and skeletal muscles using an RNA extraction kit (Guangzhou Magen Biotechnology Co., Ltd, China) and Trizol reagent (Invitrogen, Carlsbad, CA, USA) according to the manufacturer’s instructions. The total RNA was retrotranscribed into cDNA by 4 × Reverse Transcription Master Mix (A0010GQ) according to the requirements of the kit. Using the designed primers (Supplementary Table S1), 2 × SYBR Green qPCR Master Mix (ROX2 Plus) (A0001-R2) was used according to the stated procedures. cDNA synthesis was performed with the Applied Biosystems QuantStudio 3 Real-Time PCR System. Results were normalized by the expression of β-Actin or GAPDH.

### Isolation and culture of murine VSMCs

The AoSMCs were cultured in F12 DMEM (GIBCO, Grand Island, NY, USA) containing 20% fetal bovine serum (FBS) at 37°C, in a humidified atmosphere containing 5% CO_2_. According to the reference from Husarek *et al* [41]., 6- to 8-week-old mice were anesthetized by intraperitoneal injection of 200 μL 4% chloral hydrate and wiped the skin with 75% alcohol. The smooth muscle tissue was separated from the aorta and cut into 1 mm^3^ size to digest for 4 h in the cocktail (type II collagenase was added to DMEM/F12 containing 20% FBS at a concentration of 1.5 mg/mL). The cocktail was resuspended with DMEM/F12 after low-speed centrifuging (800 rpm, 4°C) and cultured in a 25 cm^2^ culture dish for 7 days, followed by sub-culturing to the third generation for treatment.

AoSMCs were treated with vehicle and 100 μmol/L AKG for 5 h after being infected with Adeno-Associated Virus-GFP for 3 days. Additionally, it would be pretreated with 50 μmol/L BAPTA-AM (HY100545, AAT Bioquest) for 1 h or 0.3 μmol/L LPA (sigma, L7260) for 10 min in treating group. The cells were placed under the fluorescence microscope to capture the different time points (0, 1, 3, and 5 h) morphology and were counted its ratio to 0 h cell morphology by Image J [42].

### Intracellular Ca^2+^ concentration assay

Intracellular Ca^2+^ concentration was measured by calcium fluorometry following the manufacturer’s instructions of fluo-8 AM kit (21080, AAT Bioquest). AoSMCs were washed twice with Hank’s Balanced Salt Solution (HBSS, pH 7.2–7.4, containing 8 g/L NaCl, 0.4 g/L KCl, 0.1 g/L MgSO_4_·7H_2_O, 0.1 g/L MgCl_2_·6H_2_O, 0.06 g/L Na_2_HPO_4_·2H_2_O, 0.06 g/L KH_2_PO_4_, 1 g/L glucose, 0.14 g/L CaCl_2_, and 0.35 g/L NaHCO_3_), followed by incubating with 10 μmol/L Fluo-8-AM at 37°C for 1 h. After incubation, cells were washed twice again. Nikon Eclipse Ti-s microscopy was used to observe fluorescence which was initiated by AKG. Fluorometric data were acquired at excitation and emission wavelengths of 490 nm and intensity at 525 nm (490/525 nm), for every 2-s interval over a 180-s period.

### Intracellular pH detection

pH_i_ was measured by pH fluorometry following the manufacturer’s instructions of BCECF-AM (117464-70-7, AAT Bioquest). AoSMCs were digested after being treated with vehicle or 100 μmol/L AKG. AoSMCs were resuspended by HEPES (pH 7.0−7.2) to incubate with 5 μmol/L BCECF-AM for 30 min. Fluorometric data were acquired at excitation and emission wavelengths of 485/528 nm and 360/528 nm, respectively.

### Statistics

Statistical analyses were performed using GraphPad Prism 8.0.1 software (Chicago, IL, USA). Methods of statistical analyses were chosen based on the design of each experiment and are indicated in the figure legends. The data are presented as mean ± SEM. *P* ≤ 0.05 was considered to be statistically significant.

## Supplementary Material

loac026_suppl_Supplementary_Material
