# Supplementary material for: AKG/OXGR1 promotes skeletal muscle blood flow and metabolism by relaxing vascular smooth muscle
Source: Life Metab. 2022 Sep 29;1(3):285–97. doi: 10.1093/lifemeta/loac026 (PMC11749495; doi:10.1093/lifemeta/loac026)
Supplement: loac026_suppl_Supplementary_Material [file loac026_suppl_Supplementary_Material.pdf]

AKG/OXGR1 Promotes Skeletal Muscle Blood Flow and Metabolism by Relaxing Vascular Smooth Muscle

Jinping Yang[1,†], Guli Xu[1,†], Yiming Xu[2], Pei Luo[1], Yexian Yuan[1], Lin Yao[3], Jingjing Zhou[1], Yunlong Zhu[1], Ishwari Gyawali[1], Chang Xu[1], Jinlong Feng[1], Zewei Ma[1], Yuxian Zeng[1], Songbo Wang[1], Ping Gao[1], Canjun Zhu[1], Qingyan Jiang[1,*] and Gang Shu[1,*]

[1] Guangdong Laboratory for Lingnan Modern Agriculture and Guangdong Province Key Laboratory of Animal Nutritional Regulation, South China Animal Nutrition and Feed Science Observation and Experimental Station, College of Animal Science, South China Agricultural University, 483 Wushan Road, Tianhe District, Guangzhou, Guangdong 510642, China.

[2] School of Basic Medical Sciences, The Sixth Affiliated Hospital of Guangzhou Medical University, Qingyuan People's Hospital, Guangzhou Medical University, Guangzhou, China.

[3] South China Research Center for Acupuncture and Moxibustion, Medical College of Acu-Moxi and Rehabilitation, Guangzhou University of Chinese Medicine, Guangzhou, 510006, China; School of Pharmaceutical Sciences, Guangzhou University of Chinese Medicine, Guangzhou, 510006, China.

[†]These authors contributed equally to this work as first author.

*Corresponding authors: Gang Shu (Lead contact, E-mail: shugang@scau.edu.cn) and Qingyan Jiang (E-mail: qyjiang@scau.edu.cn)

Supplementary Figure. S1. The validation of OXGR1-GKO and skeletal muscle OXGR1 colocalization staining. (a) OXGR1-GKO allele was identify using PCR-base genotyping. (b-c) Immunoblots of OXGR1 protein in skeletal muscle of littermate control (*n* = 6) and OXGR1-GKO (*n* = 6) mice. (d) Co-immunolocalization of OXGR1 (green) and vascular smooth muscle cells (α-SMA in red) in skeletal muscle of male littermate control (*n* = 3) and OXGR1-GKO (*n* = 4) mice. Data information: Results are presented as mean ± SEM. In (c), differences between groups were analyzed for statistical significance by Student's unpaired t test; ***P < 0.001.

Supplementary Figure. S2. Effects of OXGR1 knockout on muscle fiber type formation and capillary density in Extensor digitorum longus and Soleus. (a) Volume measurement images in Soleus. (b-c) The laminin immunofluorescent staining and soleus muscle average area statistical analysis of littermate control (*n* = 4) and OXGR1-GKO (*n* = 5) mice. (d-f) Representative images and co-staining of laminin (green) and MyHC I or MyHC IIb (red) in Extensor digitorum longus (*n* = 3) and Soleus (*n* = 4). and statistical analysis of littermate control and OXGR1-GKO mice. (g-i) Representative images and quantification of capillary (red) and arteriole (merge) immunofluorescent staining in Extensor digitorum longus (*n* = 4) and Soleus (*n* = 4) of littermate control and OXGR1-GKO mice. The Scale bar in (b, d and g) represents 50 µm. Full-field images were retained for each sample. A sample mean from 2 fields of statistical view, one of which was selected for display. Data information: Results are presented as mean ± SEM. In (c, e, f, h and i), differences between groups were analyzed for statistical significance by Student's unpaired t test; *P < 0.05, **P < 0.01.

Supplementary Figure. S3. OXGR1-GKO knockout impairs skeletal muscle slow-fiber type composition. (a-b) The SDH staining in gastrocnemius and statistical analysis of 10 weeks male littermate control (*n* = 3) and OXGR1-GKO (*n* = 3) mice. The Scale bar in (a) represents 50 µm. (c-e) Immunoblots and quantification of MyHC

I and MyHC IIa protein in gastrocnemius of littermate control (*n* = 3) and OXGR1-GKO (*n* = 3) mice. (f-h) Immunoblots and quantification of MyHC IIx and MyHC IIb protein in gastrocnemius of littermate control (*n* = 3) and OXGR1-GKO (*n* = 3) mice. Data information: Results are presented as mean ± SEM. In (b, d, e, g and h), differences between groups were analyzed for statistical significance by Student's unpaired t test; $^{*}P < 0.05$, $^{**}P < 0.01$.

Supplementary Figure. S4. Effects of dietary AKG on the muscle hypertrophy, slow muscle fiber transformation and capillary/arteriole density in extensor digitorum longus and soleus. (a-c) The laminin immunofluorescent staining in extensor digitorum longus (*n* = 6) and soleus (*n* = 7), and muscle average area statistical analysis of control and AKG groups of mice. (d-f) Representative images and co-staining of laminin (green) and MyHC I or MyHC IIb (red) in extensor digitorum longus (*n* = 4) and soleus (*n* = 4), and statistical analysis of control and AKG groups of mice. (g-i) Representative images and quantification of capillary (red) and arteriole (merge) immunofluorescent staining in extensor digitorum longus (*n* = 4) and soleus (*n* = 4) of 12-week-old control and AKG groups of mice. Scale bar in (a, d and g) represents 50 μm. Full-field images were retained for each sample. A sample mean from 2 fields of statistical view, one of which was selected for display. Data information: Results are presented as mean ± SEM. In (b and c), different between groups were analyzed for statistical significance by two-way ANOVA followed by post hoc Bonferroni tests. In (e, f, h and i), differences between groups were analyzed for statistical significance by Student's unpaired t test; $^{*}P < 0.05$, $^{**}P < 0.01$.

Supplementary Figure. S5. AKG promotes the skeletal muscle conversion of slow-fiber type. (a-b) The SDH staining in gastrocnemius and statistical analysis of 12-week-old control and AKG groups of mice (*n* = 3). The Scale bar in (a) represents 50 μm. (c-k) Immunoblots and quantification of MyHC I, MyHC IIa, MyHC IIx and MyHC IIb protein in gastrocnemius of 12-week-old control and AKG groups of mice (*n* = 3). Data information: Results are presented as mean ± SEM. In (b, d, f, i and k),

differences between groups were analyzed for statistical significance by Student's unpaired t test; **P < 0.01.

Supplementary Figure. S6. Effect of acute AKG administration on blood flow of lower limbs in adrenalectomized mice. (a-b) Representative laser speckle perfusion images and quantification of blood perfusion in lower limbs of sham and ADX mice at 5 h after being injected with physiological saline (*n* = 4) or 100 mg/kg AKG (*n* = 4). The blood flow ratio of each sample was compared with the blood flow basal value before self-injection (0 h). Data information: Results are presented as mean ± SEM. In (b), different between groups were analyzed for statistical significance by one-way ANOVA followed by by Fisher's LSD tests, **P < 0.01.

Supplementary Figure. S7. Genotyping of FloxP and Cre knock-in and schematic experimental strategy of smooth muscle-specific OXGR1 knockdown. (a) Schematic of generation of OXGR1 specific-knockdown mice. (b) OXGR1-$^{flox/flox}$ mice and MYH11-^{Cre} mice were identify using PCR-base genotyping. (c-d) Immunoblots of OXGR1 protein in skeletal muscle of OXGR1$^{MYH11+/+}$ (*n* = 6) and OXGR1$^{MYH11-/-}$ (*n* = 6) mice. (e) Co-immunolocalization of OXGR1 (green) and vascular smooth muscle cells (α-SMA in red) in skeletal muscle of female OXGR1$^{MYH11+/+}$ (*n* = 3) and OXGR1$^{MYH11-/-}$ (*n* = 4) mice. Data information: Results are presented as mean ± SEM. In (c), differences between groups were analyzed for statistical significance by Student's unpaired t test; ***P < 0.001.

Supplementary Figure. S8. The time and dose dependent relaxation effect of AKG on vascular smooth muscle cells. (a-b) Representative images and quantification of ratio of surface area at 0 h, 1 h, 3 h and 5 h, and treated with vehicle, 10 μM AKG and 100 μM AKG in primary aortic smooth muscle cells from WT mice (*n* = 3). The surface area ratio of each sample was compared with the surface area value before treatment (0 h).

Data information: Results are presented as mean ± SEM. In (b), different between groups were analyzed for statistical significance by two-way ANOVA followed by post hoc Bonferroni tests, *P < 0.05.

Supplementary Figure. S9. The relaxation effect of AKG on vascular smooth muscle cells after disturbing Ca^{2+} homeostasis and intracellular acid-base balance.

(a-b) Representative images and quantification of ratio of surface area at 0 h and 5 h, and treated with vehicle, 100 μM AKG, BAPTA-AM and BAPTA-AM with100 μM AKG in primary aortic smooth muscle cells from WT mice (*n* = 3). (c-d) Immunoblots and quantification of p-MLC/MLC protein treated with vehicle, 100 μM AKG, BAPTA-AM and BAPTA-AM with100 μM AKG in primary aortic smooth muscle cells from WT mice (*n* = 3). (e-f) Representative images and quantification of ratio of surface area at 0 h and 5 h, and treated with vehicle, 100 μM AKG, LPA and LPA with100 μM AKG in primary aortic smooth muscle cells from WT mice (*n* = 3). (g-h) Immunoblots and quantification of p-MLC/MLC protein treated with vehicle, 100 μM AKG, LPA and LPA with100 μM AKG in primary aortic smooth muscle cells from WT mice (*n* = 3). Data information: Results are presented as mean ± SEM. In (b and f), different groups were analyzed for statistical significance by one-way ANOVA followed by post hoc Dunnett's tests; In (d and h), different groups were analyzed for statistical significance by one-way ANOVA followed by Fisher's LSD tests, *P < 0.05, **P < 0.01, ***P < 0.001.

Supplementary Figure. S1

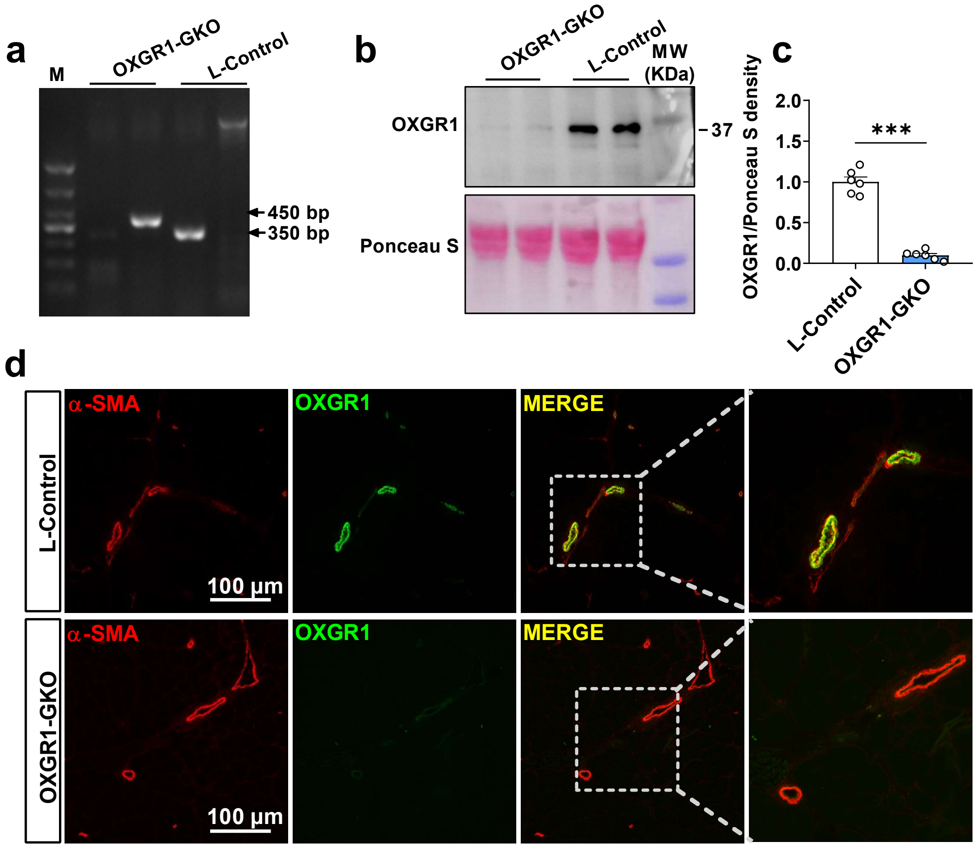

Supplementary Figure. S2

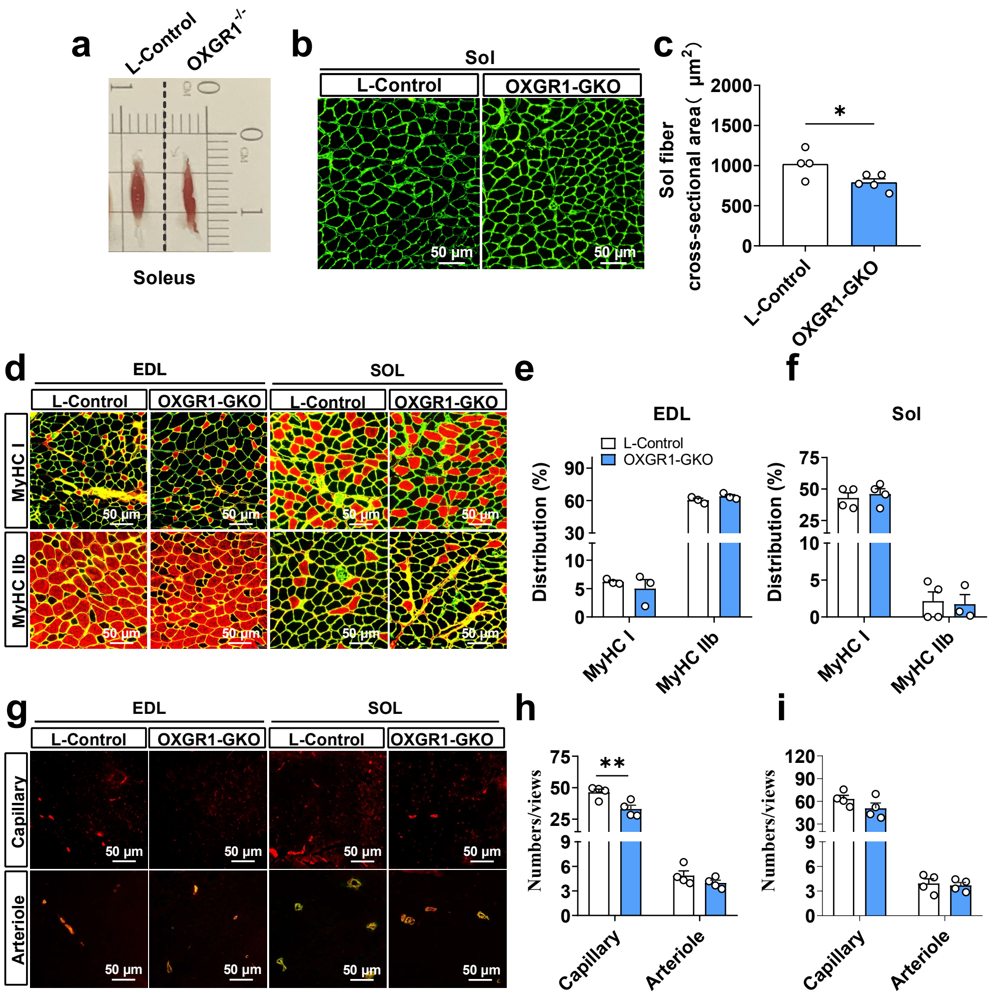

Supplementary Figure. S3

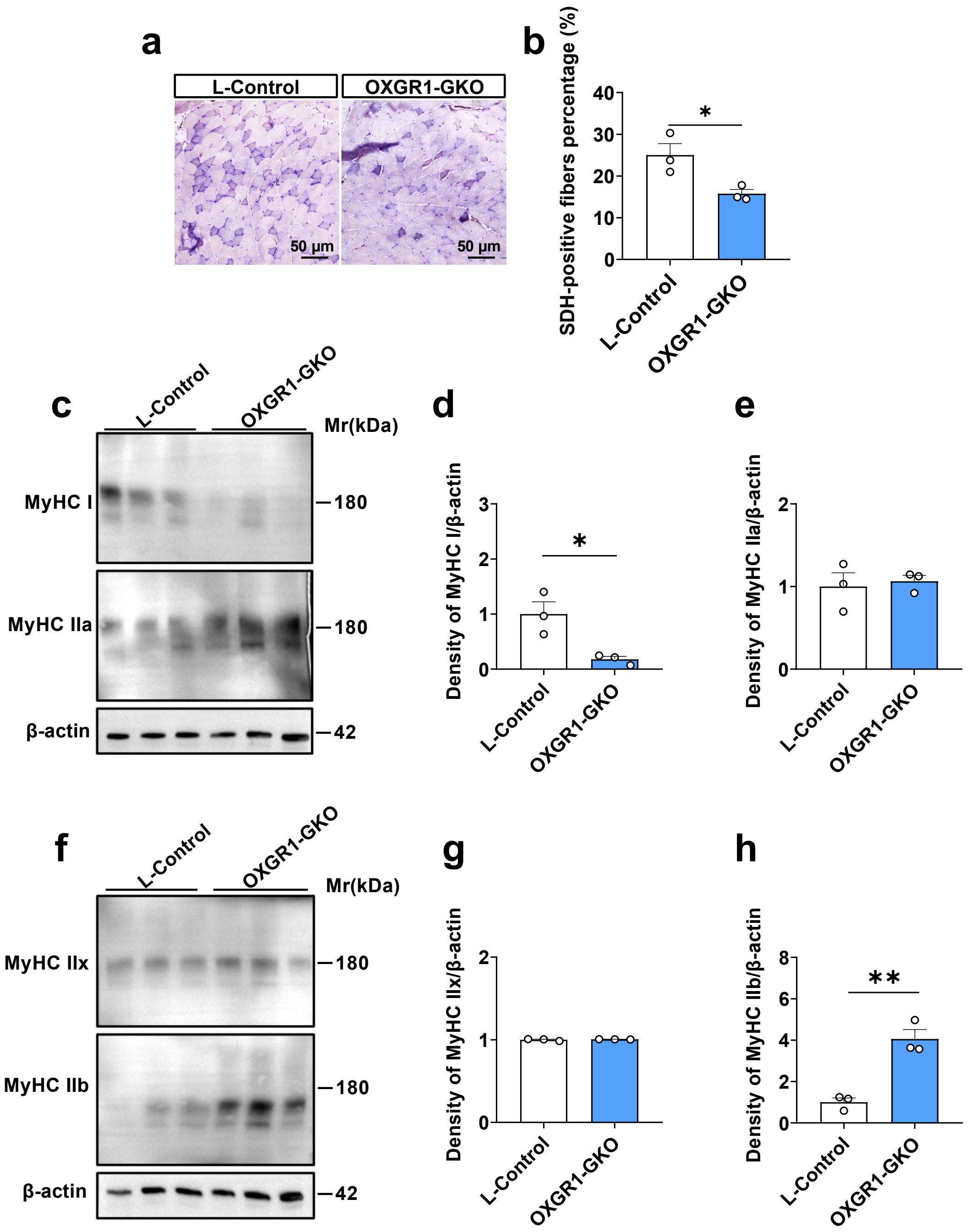

Supplementary Figure. S4

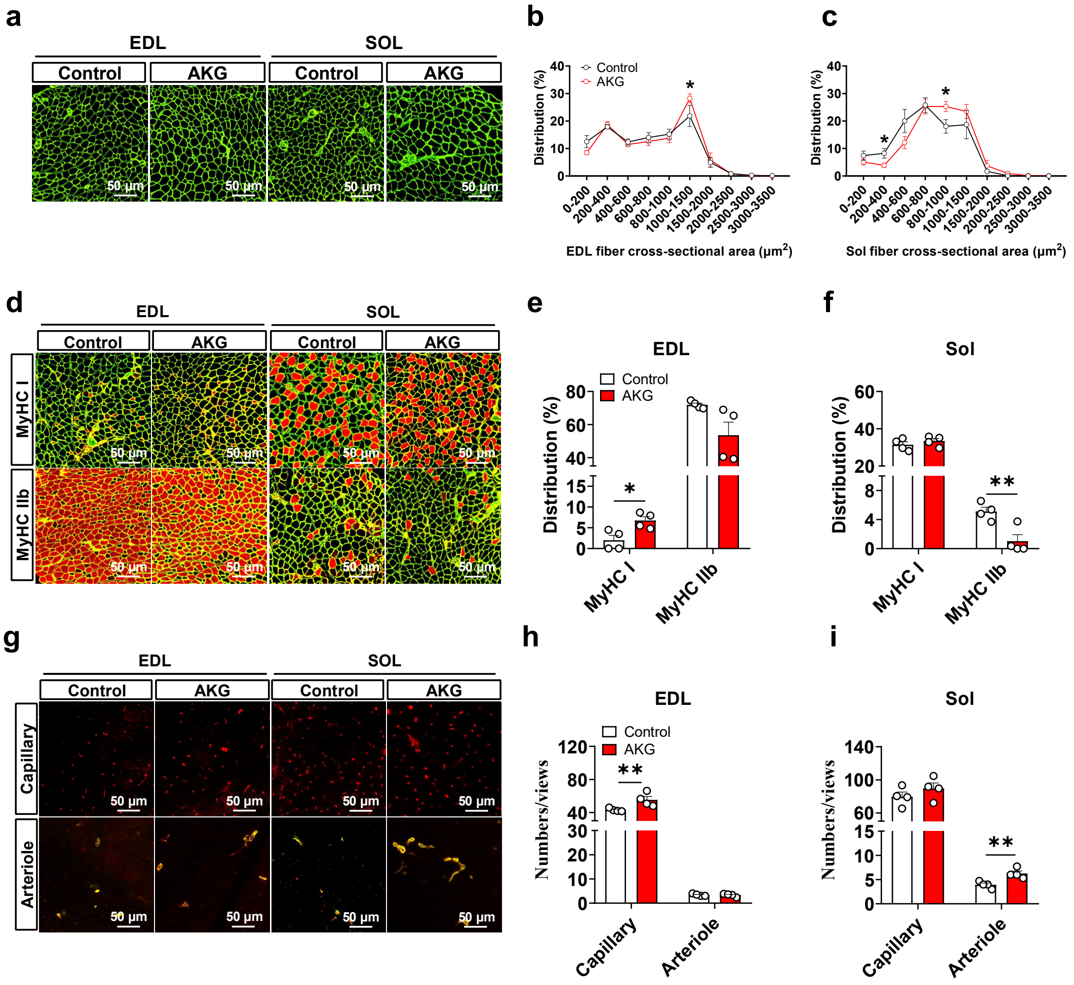

Supplementary Figure. S5

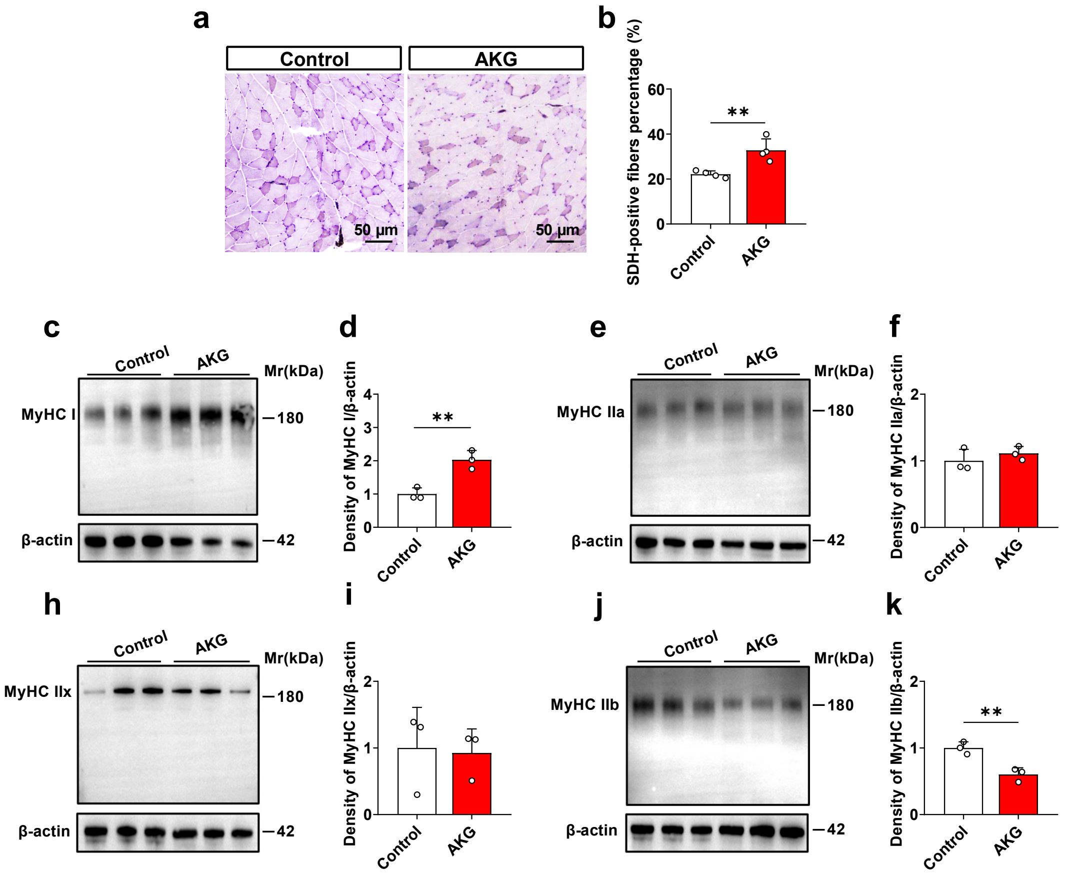

Supplementary Figure. S6

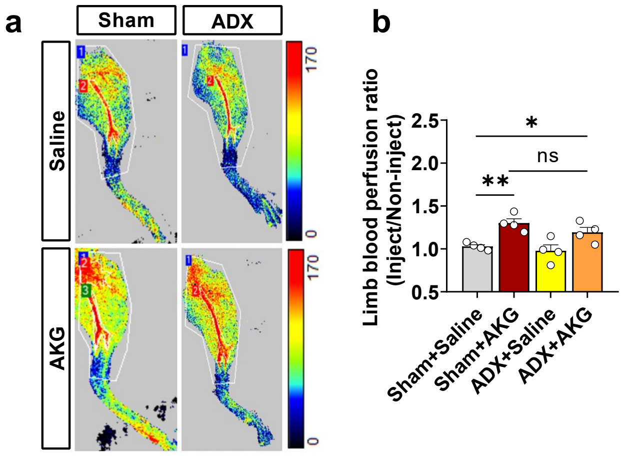

Supplementary Figure. S7

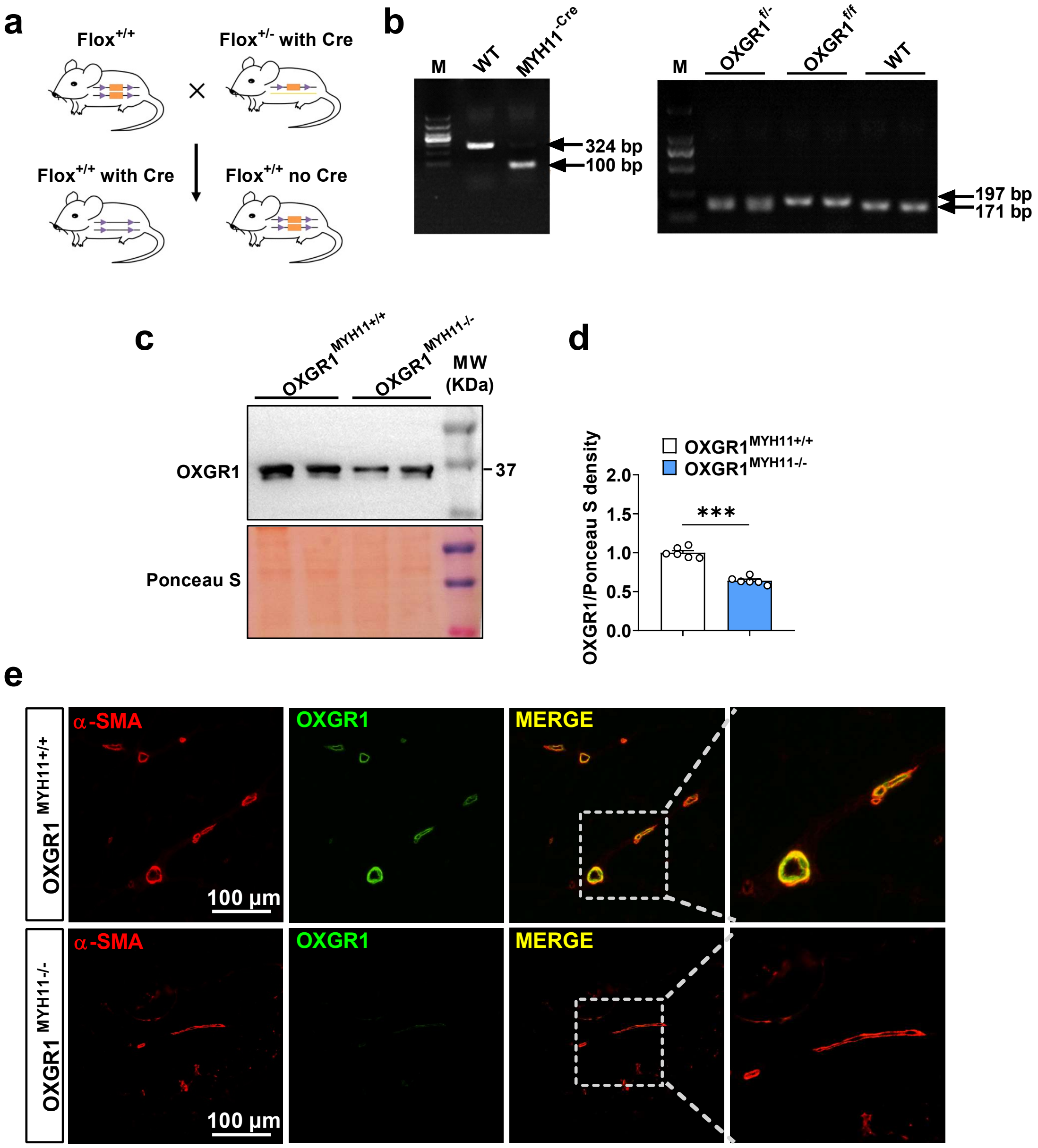

Supplementary Figure. S8

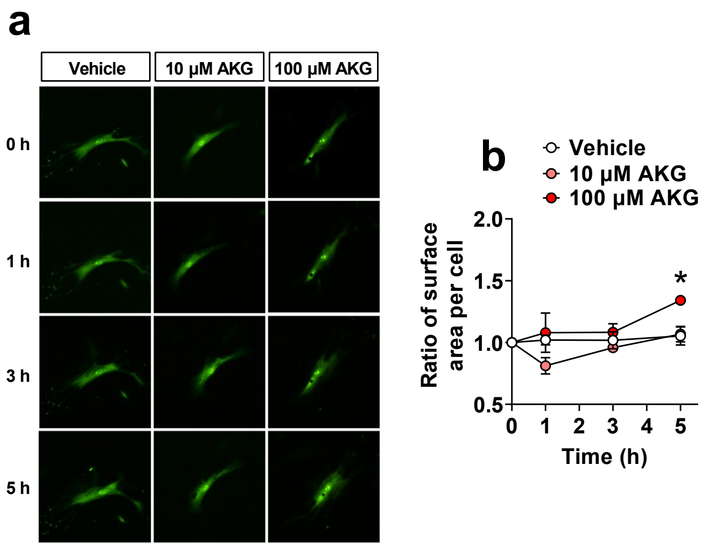

Supplementary Figure. S9

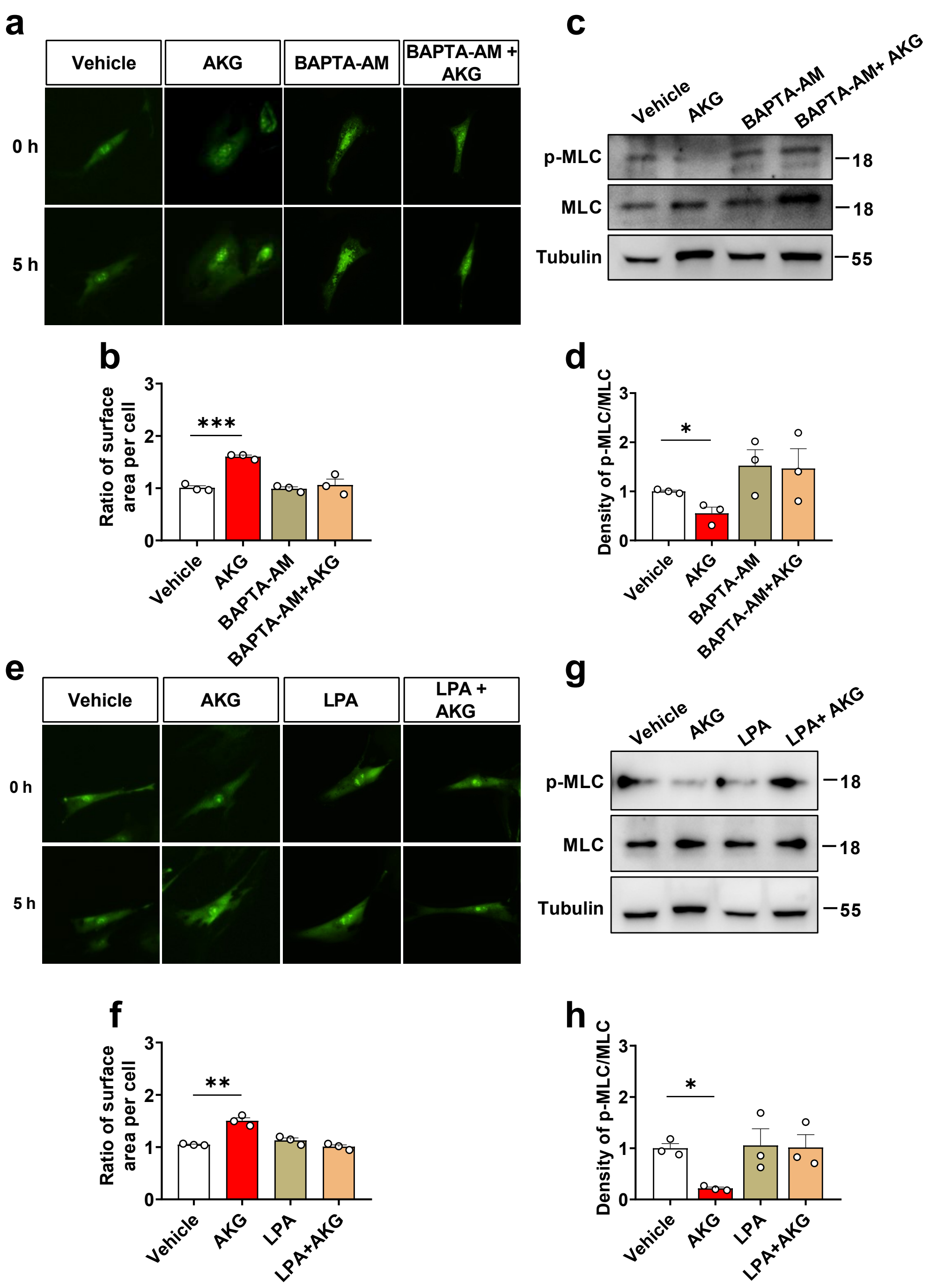

Supplementary Table. S1 PCR primer sequences of acid/base transport-related genes.

Gene	Forward (5'-3')	Reverse (5'-3')
CD31	AGTCAGAGTCTTCCTTGCCC	TCTGTTTGGCCTTGGCTTTC
VEGFR2	ACGAGGAGAGAGGGTCATCT	CAACAGGGACACACTCTCCT
MYH11	ACAGGAGGCCAGAGAGAAAC	GTACTGCTCTGCCATCTTGC
TAGLN	GGTGTGGCTGAAGAATGGTG	TTGAGCCACCTGTTCCATCT
ACTA2	GCTATTCAGGCTGTGCTGTC	GGTAGTCGGTGAGATCTCGG
SMTN	CCTCCCACAAGAAGCAGAGA	ATTCTGCTCTCTGGTTGCCT
GAPDH	AGGTCGGTGTGAACGGATTTG	TGTAGACCATGTAGTTGAGGTCA
β-actin	GGACTTCGAGCAGGAGATGG	AGGAAGGAGGGCTGGAAGAG
